# Integrated Amino Acid Profiling and 4D-DIA Proteomics Reveal Protein Quality Divergence and Metabolic Adaptation in *Cordyceps* Species

**DOI:** 10.3390/jof11050365

**Published:** 2025-05-08

**Authors:** Chuyu Tang, Yuejun Fan, Tao Wang, Jie Wang, Mengjun Xiao, Min He, Xiyun Chang, Yuling Li, Xiuzhang Li

**Affiliations:** 1 State Key Laboratory of Plateau Ecology and Agriculture, Qinghai Academy of Animal and Veterinary Sciences, Qinghai University, Xining 810016, China; chuyutang0410@163.com (C.T.); fanyuejun_79@163.com (Y.F.); 13085500761@163.com (T.W.); wangjie08142023@163.com (J.W.); 15574237597@163.com (M.X.); himi1228@163.com (M.H.); 2 The Department of Pharmacy, Qinghai Institute of Health Sciences, Xining 810016, China; 15909715156@163.com

**Keywords:** *Cordyceps sinensis*, *Cordyceps militaris*, *Cordyceps cicadae*, 4D-DIA proteomic, amino acid

## Abstract

To explore the differences in protein quality among classic medicinal entomopathogenic fungi and to evaluate their metabolic adaptability, we analyzed the amino acid composition and proteomic characteristics of *Cordyceps sinensis* (CS), *Cordyceps militaris* (CM), and *Cordyceps cicadae* (CC). Quantitative analysis showed CM contained the highest crude protein and lysine, methionine, threonine, and valine. CS adapted to high-altitude hypoxia and exhibited lower protein but elevated leucine, isoleucine, and histidine contents, which may contribute to membrane stabilization and oxidative stress resistance. CC displayed higher non-essential amino acids such as arginine, proline, and tyrosine, reflecting active nitrogen metabolism. Four-dimensional data-independent acquisition (4D-DIA) proteomics identified 495 differentially expressed proteins (DEPs). Compared with CS, CM and CC displayed upregulated glutamate oxaloacetate transaminases 2 (GOT2), glutamate dehydrogenase (GDH), and argininosuccinate synthase 1 (ASS1) coordinately regulate nitrogen flux through the alanine-aspartate-glutamate metabolic network and urea cycle, supporting metabolic intermediate replenishment for energy metabolism. The upregulation of branched-chain keto acid dehydrogenase E1 subunit alpha (BCKDHA) and acyl-CoA dehydrogenase short/branched chain (ACADSB) in CM and CC facilitated the integration of branched-chain amino acid catabolism with the TCA cycle, explaining species-specific differences in protein content. This study presents the first application of 4D-DIA proteomics to compare CS, CM, and CC, providing insights into quality divergence mechanisms in medicinal fungi.

## 1. Introduction

Medicinal and edible fungi have garnered significant attention in ethnopharmacological research and contemporary pharmacotherapy development due to their biosynthetic capacity to produce unique secondary metabolites [1]. Previous studies have revealed that medicinal fungi contain substantial quantities of structurally diverse molecules [2], including polysaccharides [3], peptides [4], phenols, purine, and immunoregulatory proteins [5], complemented by essential micronutrients such as tocopherol derivatives, ergosterol precursors, and bioavailable mineral complexes [6]. Therefore, the fungi mediate their therapeutic efficacy by exhibiting bioactive properties, representing an underexploited source for nutraceutical development. Among these components, fungal proteins have emerged as a significant alternative protein source, offering a sustainable and versatile approach to addressing global food and nutrition security challenges [7]. In contrast to traditional meat- and legume-based proteins, the nutritional composition of fungal proteins and characteristic secondary metabolites produced under stress conditions may be pivotal in exerting their therapeutic effects [8]. Prior pharmacological research suggested medicinal fungal proteins exhibit distinct therapeutic advantages through their targeted mechanisms and multifunctional properties [9]. Lin et al. demonstrated that fungal immunomodulatory proteins LZ-8 and GMI isolated from *Ganoderma lucidum* and Ganoderma microsporum inhibit lung cancer cell migration and induce apoptosis by downregulating heat shock proteins (HSPs) 60, 70, and 90, with their anti-tumor activity attributed to the modulation of HSP-mediated protein degradation pathways [10]. The protein-bound galactomannan component isolated from the extracellular polysaccharides of *Cordyceps sinensis* Cs-HK1 exhibits strong antioxidant properties, and it can significantly protect PC12 cells from H_2_O_2_-induced cell death within the concentration range of 50 to 250 μg/mL [11]. Additionally, Kong et al. demonstrated that *Ophiocordyceps sinensis* OCS02 exhibits bioactive proteins, including cytotoxic proteases and nephroprotective oxalate decarboxylase, further underscoring its pharmacological potential [12].

The genus *Cordyceps* constitutes the most species-rich group within the Clavicipitaceae family [13]. As ecologically specialized fungi, interspecific variation is primarily defined by distinct geographical distributions and host-specific parasitism on targeted insect species [14]. These fungi demonstrate significant biodiversity, with a broad biogeographical distribution concentrated in East and Southeast Asia, notably across China, Japan, Nepal, Vietnam, and Bhutan [15]. Taxonomic records currently document over 750 identified *Cordyceps* species, with over 30 species demonstrating confirmed pharmacological properties [16]. Notably, *Cordyceps sinensis*, *Cordyceps militaris*, *Cordyceps cicadae* [17], and *Cordyceps guangdongensis*, alongside their fermentation-derived mycelial products [18], have been commercialized as dietary supplements and nutraceuticals, garnering substantial scientific and industrial interest [1]. Among these, *C. sinensis*, *C. militaris*, and *C. cicadae* have emerged as the most intensively researched taxa due to their evolutionarily adapted survival strategies [13]. This can be attributed to their specialized survival mechanisms, which elicit host defense responses and subsequently result in the biosynthesis of diverse primary metabolites and distinctive secondary metabolites [19]. Pharmacological investigations have identified these three species as rich sources of bioactive compounds, including polysaccharides, amino acids, nucleotides, and low-molecular-weight peptides [20], contributing to therapy for respiratory diseases, cardiovascular pathologies, and metabolic disorders [21].

Technology platforms integrating liquid chromatography (LC) with data-independent acquisition (DIA) mass spectrometry (MS) have emerged as widely adopted solutions for quantitative proteomics analysis [22]. Notably, DIA demonstrates superior accuracy, efficiency, and reproducibility compared to traditional proteomics quantification methods and has found extensive applications in biomarker discovery [23], clinical research [24], and food science [25]. Currently, research on the processing techniques, pharmacological activities, and domestication of strains related to medicinal fungi has increasingly become a focal point in scientific investigations [14]. However, research focusing on their proteins and the correlations between these proteins and quality attributes remains relatively limited. Previous studies have revealed the metabolic profiles of four species of *Cordyceps* and have demonstrated that the Maximum Entropy (MaxEnt) model can be utilized to predict the habitat-suitability distribution of the *Cordyceps* [19]. Furthermore, it was identified that lipid-related compounds constituted the primary metabolites, potentially indicating a close association with the organisms’ living environments [16]. Indeed, proteins not only encode the functional adaptive traits that enable fungi to survive in complex ecosystems [26] but also serve as crucial resources for developing novel biological agents due to their high specificity and diverse pharmacological properties. Therefore, 4D-DIA proteomics technology was employed in this study to assess the proteomic differences among three medicinal entomopathogenic fungi. Additionally, quantitative analysis of amino acid contents was integrated to elucidate the underlying reasons for variations in protein quality across different medicinal fungi. This study not only enhances our understanding of proteomic changes in *Cordyceps* but also aids in identifying and characterizing signature proteins and potential therapeutic targets.

## 2. Materials and Methods

### 2.1. Materials

*C. militaris* (CM) and *C. cicadae* (CC) were purchased from Qinghai Baohuitang Bio-technology Co., Ltd. (Xining, China). CM specimens were originally collected from Shenyang, Liaoning Province, China (41°51′25″ N, 123°19′2″ E), and CC specimens were collected from Huoshan County, Liuan City, Anhui Province, China (31°07′25.7″ N, 116°11′38.3″ E). *C. sinensis* (CS) was obtained from Qinghai Qingqitang Trading Co., Ltd. (Xining, China), with a collection site in Zaduo County, Yushu City, Qinghai Province, China (33°08′15″ N, 95°38′12″ E). All samples were stored in an ultra-low-temperature freezer at −80 °C pending subsequent analytical procedures.

### 2.2. Determination of Crude Protein and Amino Acid

Protein content was detected by the national food safety standard (GB_T5009.5-2016) [27]. The crude protein content was determined by sulfuric acid digestion and the Kjeldahl nitrogen determination method [28]. For each indicator, three biological replicates were established to ensure the reliability and consistency of the results.

The absolute quantitative analysis of 20 amino acids, including alanine, valine, and leucine, isoleucine, glutamic acid, lysine, arginine, glycine, serine, threonine, cysteine, aspartic acid, asparagine, glutamine, phenylalanine, tyrosine, tryptophan, histidine, proline, and methionine, was performed based on the standard curve (Figure S1). Twenty standard substances were dissolved in ultrapure water to prepare a 1000 nmol/mL standard stock solution. Subsequently, the standard stock solution was serially diluted with ultrapure water to obtain a series of working standard solutions with concentrations of 0.01, 0.05, 0.1, 1, 5, 10, 20, and 50 nmol/mL. Finally, the standard curve was constructed based on these solutions, as presented in Figure S1. The chromatographic conditions were as follows: an Agilent Poroshell 120 HILIC-Z column (2.7 μm, 3.0 × 150 mm) was used; the column temperature was set at 30 °C; the flow rate was maintained at 0.5 mL/min; the injection volume was 5 μL; mobile phase A consisted of a 0.1% formic acid aqueous solution; mobile phase B was acetonitrile; and gradient elution was performed. The mass spectrometry parameters were as follows: electrospray ionization (ESI) in positive ion mode was employed; the scan type was multiple reaction monitoring (MRM); the source/gas parameters were as follows: Curtain Gas (CUR), 35.0 psi; IonSpray Voltage (IS), 5500 V; Temperature (TEM), 500 °C; Ion Source Gas 1 (GS1), 60.0 psi; Ion Source Gas 2 (GS2), 60.0 psi.

### 2.3. Proteomics Profiling

All *Cordyceps* specimens have three independent biological replicates. Protein samples were made by blending tissues in a lysis solution (8 M urea, 1% SDS, protease inhibitors) with three 40 s grinding cycles. The mixture was incubated at 0–4 °C for 30 min with vortexing. After centrifugation (16,000× *g*, 4 °C, 30 min), protein concentration in the supernatant was measured using a BCA kit (Thermo Fisher Scientific, Shanghai, China).

For each 100 μg protein aliquot, it was dissolved in 100 mM TEAB buffer, reduced with 10 mM Tris (2-carboxyethyl) phosphine at 37 °C for 1 h, then alkylated with 40 mM iodoacetamide in the dark at room temperature for 40 min. Protein precipitation was performed by adding ice-cold acetone (6:1 *v*/*v*) and storing at −20 °C for 4 h. After centrifugation (10,000× *g*, 20 min), the pellet was resuspended in 100 µL 100 mM TEAB and digested with trypsin (1:50 enzyme-to-protein) at 37 °C for 12–16 h. Peptides were desalted and quantified using a commercial kit (23275, Thermo Fisher Scientific) [29]. Equal peptide amounts were combined, concentrated by vacuum centrifugation, and resuspended in UPLC loading buffer (Phase A: 2% acetonitrile, pH 10; Phase B: 80% acetonitrile, pH 10). To enhance proteomic depth, the mixed peptides were fractionated using a Vanquish Flex binary UHPLC system with an Acquity UPLC BEH C18 Column (1.7 µm, 2.1 mm × 150 mm). The 47 min gradient elution at 200 μL/min was programmed as follows: 0–16 min, 0% B; 16–17 min, 0–3.8% B; 17–34 min, 3.8–24% B; 34–37 min, 24–30% B; 37–38 min, 30–43% B; 38–39 min, 43–100% B; 39–44 min, 100–0% B; and 44–47 min, 0% B. Six fractions were collected.

DIA mass detection was performed using a timsTOF Pro2 mass spectrometer in DIA-PASEF mode. The C18 column (75 μm × 25 cm) was equilibrated with solvent A (2% ACN, 0.1% formic acid) and solvent B (80% ACN, 0.1% formic acid). Peptides were eluted with a gradient: 0–45 min, 3–28% B; 45–50 min, 28–44% B; 50–55 min, 44–90% B; 55–60 min, 90% B, at 250 nL/min. Data were processed using Spectronaut software (v14.10.201222.47784; Biognosys) against a custom protein database derived from the reference genome GCA_012934285.1_ASM1293428v1 (NCBI: https://ftp.ncbi.nlm.nih.gov/genomes/all/GCA/012/934/285/GCA_012934285.1_ASM1293428v1/, accessed on 10 December 2024). Search parameters included the following: peptide length of 7–52 amino acids, trypsin/P specificity with up to two missed cleavages, fixed carbamidomethylation (C), variable oxidation (M) and N-terminal acetylation, 10 ppm precursor mass tolerance, 20 mmu fragment mass error, and a 1% protein FDR threshold. Protein quantification utilized the MaxLFQ algorithm, integrating unique/shared peptides via iterative optimization of peptide-to-protein ratios while excluding homologous-conflicting peptides, with final results filtered at ≤1% FDR for both peptides and proteins to ensure reliability.

### 2.4. Bioinformatics Analysis

The proteomic data obtained were subjected to bioinformatic analysis using the Majorbio Cloud Platform (https://cloud.majorbio.com, accessed on 28 December 2024). Differential protein expression was analyzed with the R package “Student’s *t*-test” (https://www.r-project.org/, accessed on 28 December 2024), considering significance thresholds of |log2 Fold Change| ≥ 1 and *p*-value < 0.05. Functional annotation was performed using Gene Ontology (GO; http://geneontology.org/, accessed on 29 December 2024) and the Kyoto Encyclopedia of Genes and Genomes pathway database (KEGG; http://www.genome.jp/kegg/, accessed on 28 December 2024). Differentially expressed proteins (DEPs) were further analyzed for GO and KEGG enrichment.

### 2.5. Statistical Analysis

All tests utilized three biological replicates. Normally distributed data were assessed via one-way ANOVA (SPSS v26.0) and visualized with GraphPad Prism (v10.0).

## 3. Results

### 3.1. Determination of Crude Protein and Amino Acids

**Figure 1:**
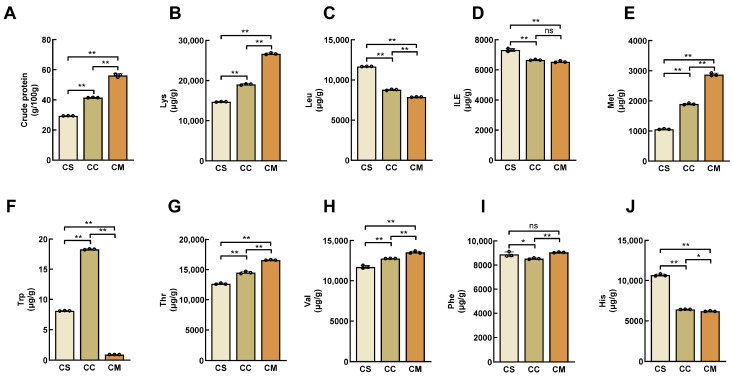
Determination of essential amino acid content. (**A**) Crude protein content; (**B**) Lysine content; (**C**) Leucine content; (**D**) Isoleucine content; (**E**) Methionine content; (**F**) Tryptophan content; (**G**) Threonine content; (**H**) Valine content; (**I**) Phenylalanine content; (**J**) Histidine content. “*” indicates a significant difference (*p* < 0.05); “**” indicates an extremely significant difference (*p* < 0.01); “ns” indicates the difference is not significant (*p* > 0.05).

Crude protein content is a crucial parameter for evaluating the nutritional quality of fungi and plays a pivotal role in quality assessment. In our study, we quantitatively analyzed the crude protein content and observed that all three Cordyceps species (CS, CC, and CM) exhibited high protein levels (Figure 1A). Specifically, the crude protein contents of CS, CC, and CM were 29.45 ± 0.08 g/100g, 41.51 ± 0.15 g/100g, and 56.23 ± 1.06 g/100g, respectively. Notably, the crude protein content of CM was approximately 1.4 times higher than that of CS, indicating its significantly richer protein composition. Furthermore, amino acid content is always a key determinant of the nutritional value of food and an essential component for normal cellular physiology and metabolism [30]. Typically, amino acids are categorized into essential amino acids (EAAs) and non-essential amino acids (NEAAs), depending on whether they can be adequately obtained from the diet to meet optimal human nutritional needs [31]. By analyzing the levels of nine EAAs (Figure 1B–J), Val, Leu, ILE, Phe, Trp, Thr, Lys, Met, and His, we observed that the CM exhibited significantly higher levels than the CS and CC. Notably, the CM demonstrated the highest concentrations of Lys, Met, Thr, and Val. Conversely, CS had the highest levels of Leu, ILE, and His compared to CM and CC, while Trp was the most abundant in CC. Then, we performed a quantitative analysis of the NEAAs in CS, CC, and CM (Figure S2). The results revealed that CS was particularly abundant in Glu, Cys, and Asn, whereas CC exhibited higher levels of Arg, Pro, Tyr, Ala, Gln, and Asp. Notably, Gly was found to have the highest content in CM at 23,388.74 ± 144.80 μg/g, surpassing the levels observed in the other two Cordyceps species. These findings suggest that while significant differences exist in the types of amino acids among various medicinal fungi, their concentrations can also vary substantially.

### 3.2. Comprehensive Analysis of Proteomic Profiles

**Figure 2:**
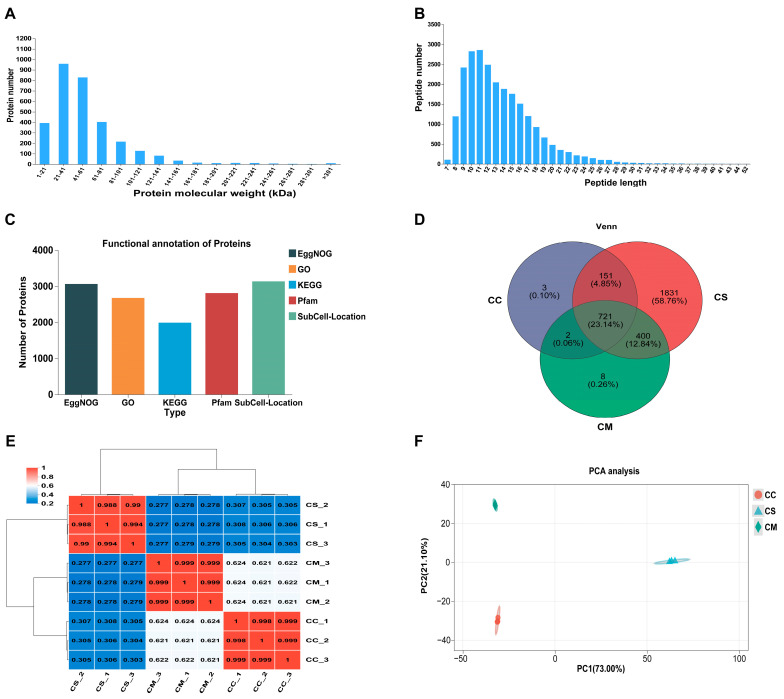
Comprehensive analysis of proteomics. (**A**) Protein molecular weight distribution; (**B**) Peptide segment length distribution; (**C**) Protein group functional annotation; (**D**) Venn analysis of all proteins; (**E**) Correlation analysis of samples; (**F**) PCA of the sample.

We conducted a proteomic analysis of the proteins identified in CS, CC, and CM. Results demonstrated that a higher number of proteins were detected within the lower molecular weight range of 21–41 kDa, with a gradual decrease in protein abundance as molecular weight increased (Figure 2A). Subsequently, we observed that peptides typically ranged from 8 to 21 amino acids in length, and the number of peptides initially increased and then decreased with increasing length (Figure 2B), suggesting high detection efficiency and sensitivity in mass spectrometry analysis and confirming the reliability of the proteomics data. Therefore, a total of 26,081 peptide segments were identified, corresponding to 3116 unique proteins, and were subjected to a comparative analysis against several databases, including GO, KEGG, EggNOG, Pfam, and subcellular localization databases. The results revealed that the highest number of proteins were annotated by EggNOG, followed by SubCell-Location. In contrast, the number of proteins annotated by KEGG was relatively lower (Figure 2C).

We analyzed the common and unique proteins among all the sample groups. It was found that 721 proteins were shared by CC, CS, and CM simultaneously, while CS, CC, and CM had 1831, 3, and 8 unique proteins, respectively (Figure 2D). Among these common proteins, three species of Cordyceps contain various proteins that are involved in metabolism, cytoskeleton, transport, and signal transduction and regulation. Among them, there are key metabolic enzymes, such as pyruvate carboxylase (PC), fumarate hydratase (FH), and dihydrolipoyl dehydrogenase (DLD), that are involved in central carbon metabolism. Additionally, deoxyuridine 5′-triphosphate nucleotidohydrolase (DUT) prevents uracil misincorporation into DNA by hydrolyzing dUTP to dUMP, while uracil phosphoribosyltransferase (UPRT) salvages uracil to sustain pyrimidine pools [32]. These enzymes collectively regulate intracellular nucleotide homeostasis, ensuring the fidelity and efficiency of RNA and DNA synthesis. For different Cordyceps, a correlation heatmap visualization was performed, which revealed high correlations within groups and low correlations between groups (Figure 2E). Meanwhile, principal component analysis (PCA) of the samples showed that PC1 (73.00%) and PC2 (21.10%) accounted for the majority of the variance and demonstrated distinct grouping trends among the samples (Figure 2F). These results indicate significant differences in protein expression profiles across the sample groups.

### 3.3. Annotation Analysis of Total Protein

**Figure 3:**
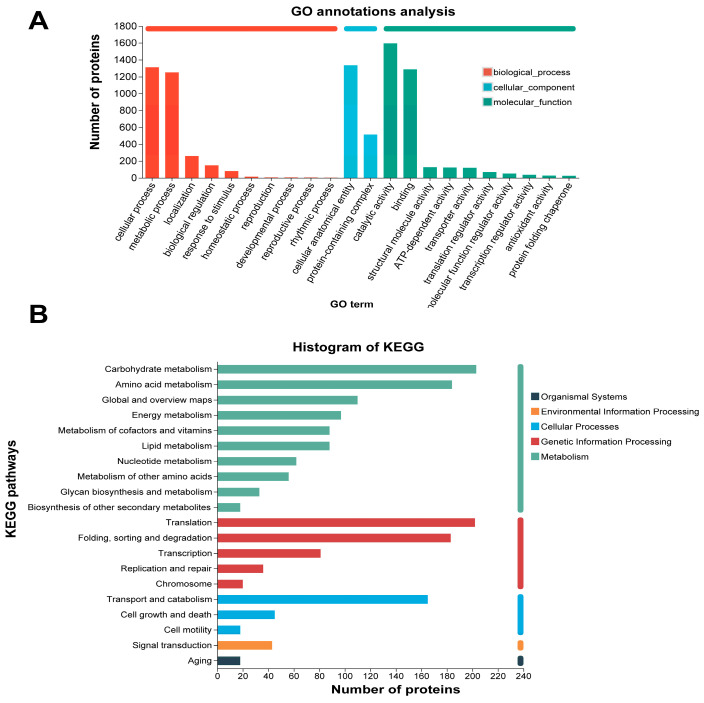
Analysis of GO and KEGG annotations for proteins. (**A**) GO annotations for proteins. The *x*-axis of the figure represents specific GO terms, while the *y*-axis indicates the corresponding number of proteins; (**B**) KEGG annotations for proteins. The *x*-axis denotes specific KEGG pathways, while the *y*-axis indicates the corresponding number of proteins.

GO annotations revealed that proteins were predominantly annotated under the GO terms cellular process, metabolic process, and response to stimulus within the biological process, suggesting their potential role in reflecting fundamental cellular activities and adaptation to environmental changes (Figure 3A). In the cellular component, a significant proportion of proteins were annotated under terms such as cellular anatomical entity and protein-containing complex. Many proteins were annotated in the molecular function category with terms like binding and catalytic activity, indicating their involvement in molecular interactions and biochemical reactions.

Subsequently, we performed a KEGG pathway analysis (Figure 3B). Our analysis revealed that a substantial number of proteins were annotated in metabolic pathways such as carbohydrate metabolism, amino acid metabolism, and lipid metabolism, suggesting their potential involvement in cellular material and energy metabolism activities. Additionally, numerous proteins were identified in genetic information processing pathways, including translation, folding, sorting, and degradation, indicating that these processes were highly active in the sample and likely associated with critical life activities such as protein synthesis, modification, and degradation. Furthermore, a notable number of proteins were annotated in pathways related to transport and catabolism, cell growth, and death, implying the presence of proteins involved in signal transmission and cellular regulation within the sample, which may contribute to the complex regulatory networks of the cell.

### 3.4. Screening and Analysis of Differential Proteins

**Figure 4:**
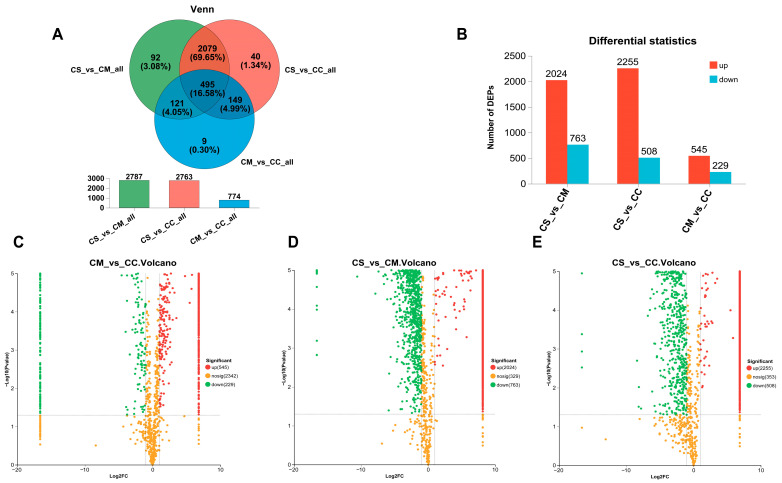
DEPs in different Cordyceps. (**A**) Venn diagram analysis of DEPs for CS, CM, and CC; (**B**) statistical analysis of DEPs; (**C**–**E**) volcano plot analysis of DEPs for CS, CM, and CC. The *x*-axis represents the log2-fold change in protein expression levels between the two samples, while the *y*-axis indicates the −log10-adjusted *p*-values reflecting the statistical significance of these differences. Each point on the plot corresponds to a specific protein. Proteins with downregulated expression are represented by points located on the left side of the plot, whereas those with upregulated expression are shown on the right.

We screened the DEPs based on |log2FC| ≥ 1 and *p*-value < 0.05. It was found that there were 495 common DEPs among CS, CC, and CM (Figure 4A). A total of 92 DEPs were identified between CS and CM, indicating a relatively high degree of similarity in their protein compositions. In contrast, only nine DEPs were detected between CM and CC, suggesting substantial differences in their protein profiles. An analysis of the number of DEPs in the comparisons of CS vs. CM, CS vs. CC, and CM vs. CC revealed that 2024 DEPs were upregulated and 763 were downregulated in CS vs. CM; 2255 DEPs were upregulated and 508 were downregulated in CS vs. CC; and 545 DEPs were upregulated and 229 were downregulated in CM vs. CC (Figure 4B). The relatively high number of DEPs observed between CS and CM, as well as between CS and CC, suggests substantial changes in protein expression levels. Notably, the number of upregulated proteins was significantly greater than that of downregulated proteins in these comparisons. In the volcano plots of the differences between DEPs in Figure 4C–E, we visualized the upregulation and downregulation patterns of DEPs. This pattern may be attributed to the long-term adaptation of CS to high-altitude and hypoxic environments under adverse stress conditions, which likely induced widespread upregulation of protein expression. In contrast, CM and CC exhibited relatively fewer changes in protein expression.

### 3.5. GO Enrichment Analysis of DEPs

**Figure 5:**
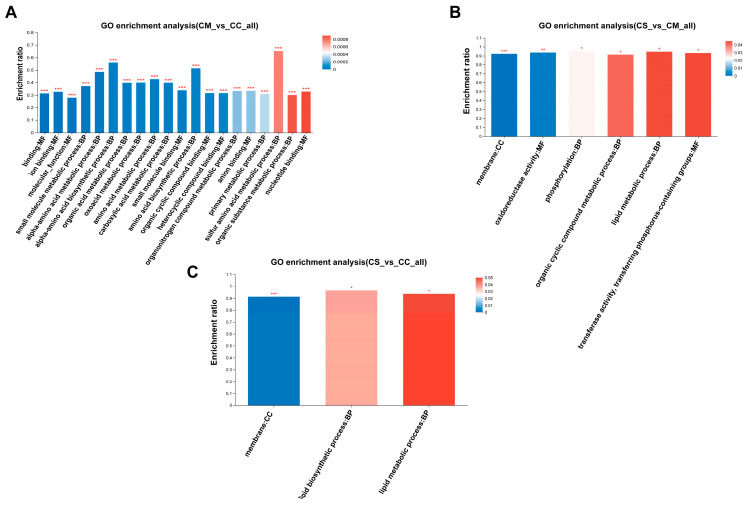
GO enrichment analysis of DEPs. (**A**) GO enrichment analysis of CM vs. CC; (**B**) GO enrichment analysis of CS vs. CM; (**C**) GO enrichment analysis of CS vs. CC. The *x*-axis represents the GO terms, while the *y*-axis represents the enrichment ratio. The column color gradient reflects the significance of enrichment. GO terms with *p* or FDR values < 0.001 are marked with “***”, those with *p* or FDR values < 0.01 are marked with “**”, and those with *p* or FDR values < 0.05 are marked with “*”.

Significant functional GO enrichment analysis was conducted on DEPs to elucidate the biological processes, cellular components, and molecular functions that proteins are involved in at the functional level. We found that in the comparison of CM vs. CC, small molecule metabolic processes, organic cyclic compound metabolic processes, α-amino acid biosynthetic processes, and catalytic activities showed significant enrichment between CM and CC (Figure 5A), indicating that these biological processes may have undergone significant changes in CM and CC, and the DEPs of these processes may affect the corresponding biochemical reactions and cellular functions. In CS vs. CM (Figure 5B), entries such as lipid metabolism processes and primary metabolism processes showed significant enrichment, suggesting that lipid metabolism processes in CS may have been activated or inhibited, which is similar to the enrichment degree in CS vs. CC (Figure 5C). Terms such as lipid synthesis processes and lipid metabolism processes showed significant enrichment, indicating that the lipid synthesis and metabolism processes in CS samples may have undergone significant changes, which may be related to biological functions such as energy metabolism and dynamic regulation of cell membranes.

### 3.6. Analysis of Enrichment Pathways for Key DEPs

**Figure 6:**
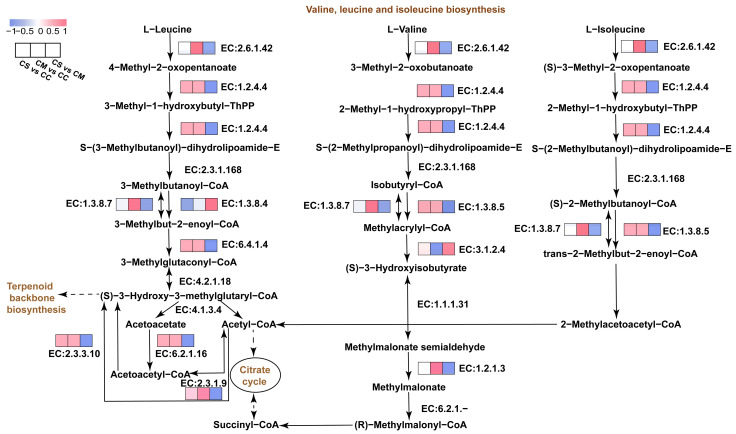
Annotation and analysis of key pathways. The heatmap is generated using Log2 (FC) values; pink indicates an increase, while blue indicates a decrease.

Based on the previous enrichment analysis of DEPs, we found that DEPs are significantly mainly involved in the amino acid biosynthesis and degradation of CS, CC, and CM. We further conducted KEGG discovery on the DEPs under the entries. Most of the DEPs are involved in the valine, leucine, and isoleucine degradation (Figure 6). Therefore, we analyzed the related pathways of the three species of Cordyceps. This pathway encompasses the degradation of branched-chain amino acids (BCAAs), including L-leucine, L-valine, and L-isoleucine. Through metabolic processes, it indirectly influences the tricarboxylic acid cycle and steroid skeleton synthesis. Specifically, EC: 2.6.1.42 (branched-chain-amino-acid aminotransferase, BCAT) catalyzes the conversion of L-leucine, L-valine, and L-isoleucine into 3-methyl-2-oxobutanoate and other corresponding 2-oxo acids. Notably, in the comparison of CM vs. CC, this enzyme is significantly upregulated compared to the other two groups, enhancing the availability of initial substrates and accelerating the entire biosynthetic pathway. Subsequently, EC: 1.2.4.4 (branched-chain keto acid dehydrogenase E1 subunit alpha, BCKDHA) catalyzes the oxidative decarboxylation of these 2-oxo acids to form their corresponding acyl-CoA derivatives. EC: 2.3.1.168 (branched-chain dihydrolipoamide acyltransferase, BCDAT), a component of the branched-chain keto acid dehydrogenase complex, further converts these α-keto acids into lipoamide thioester derivatives by transferring acyl groups to coenzyme A, generating acyl-CoA. These acyl-CoA derivatives are then subjected to β-oxidation via the actions of EC: 1.3.8.7 (medium-chain acyl-CoA dehydrogenase, ACADM) and EC: 1.3.8.5 (branched-chain acyl-CoA dehydrogenase, ACADSB), which catalyze the dehydrogenation step in fatty acid catabolism. Concurrently, EC: 3.1.2.4 (3-hydroxyisobutyryl-CoA hydrolase, HIBCH) converts 3-hydroxyisobutyryl-CoA into methylacrylyl-CoA. Through a series of enzymatic reactions, 2-methylacetoacetyl-CoA is converted into acetyl-CoA and participates in the tricarboxylic acid cycle. For L-isoleucine, its catabolic pathway follows similar steps and involves EC: 2.3.1.9 (acetyl-CoA acetyltransferase, ACAT) in downstream processing. These pathways are intricately linked to the TCA cycle via acetyl-CoA and succinyl-CoA, and they intersect with steroid skeleton biosynthesis through shared metabolic intermediates.

## 4. Discussion

As typical medicinal entomopathogenic fungi, CS, CC, and CM exhibit various pharmacological activities that rely on protein composition. Previous studies have systematically investigated the metabolic profiles of CM, CS, and CC, demonstrating that these three species are abundant in both primary and secondary metabolites. The quality variations among them may be closely associated with amino acid metabolism and lipid metabolism [16]. However, the underlying mechanisms of action of key active components, such as proteins and amino acids, remain to be further elucidated. The quantity of proteins and the diversity of hydrolyzed amino acids serve as critical determinants of their quality. Through quantitative analysis of crude protein and 20 hydrolyzed amino acids, we identified significant differences among the three *Cordyceps* species. Through quantitative analysis, we found that CM contains higher levels of crude protein and hydrolyzed amino acids compared to other species. Specifically, Lys, Met, Thr, and Val exhibit the highest concentrations in CM. The observed disparity may stem from distinct host larval characteristics between CM, CC, and CS. Specifically, CM enhances nutrient assimilation during larval development and promotes both mycelial formation and protein enrichment through superior host-derived nutritional provisioning [33]. In contrast, CS, having long adapted to high-altitude hypoxic environments with its substrate restricted to specific host larvae, exhibits reduced protein content due to the absence of exogenous carbon-nitrogen supplementation [34]. According to the research findings of Wang et al., as altitude increases, ecological factors influencing CS, such as temperature, oxygen concentration, and radiation intensity, compel CS to evolve survival strategies to counteract low temperatures and ultraviolet radiation [35]. Furthermore, a previous study also demonstrates that different plant species mitigate high-altitude abiotic stress through the modulation of photosynthesis, regulation of leaf cell osmotic pressure, and activation of various oxidative protective mechanisms [36]. Interestingly, CS demonstrates higher levels of Leu, Ile, and His compared to CC and CM. This phenomenon may elucidate the protective mechanism developed by CS in response to prolonged exposure to low temperatures and intense ultraviolet radiation in high-altitude environments. Specifically, it facilitates the synthesis of certain free amino acids, which enhances adaptability to high-altitude conditions. These findings are consistent with those reported in prior studies [37]. Additionally, the prolonged exposure of CS to high-altitude ultraviolet radiation may enhance the production of reactive oxygen species (ROS) [38], leading to an increased content of histidine. As a precursor for antioxidants, histidine plays a critical role in scavenging ROS and alleviating oxidative stress induced by prolonged ultraviolet exposure [39]. By comparison, CM and CC grow in milder environments, reducing their demand for these protective amino acids. Our analysis of non-essential amino acids demonstrated that CC exhibited significantly higher levels of Arg, Pro, Tyr, Ala, Gln, and Asp compared to CS and CM. Arginine serves as a critical intermediate in nitrogen metabolism, decomposing into ornithine, which subsequently converts into glutamate and acts as a precursor for other amino acids, promoting the accumulation of amino acids. Consequently, arginine plays a pivotal role in maintaining normal amino acid metabolic pathways. Furthermore, given that the habitats of CC and CM are not exposed to adverse environmental pressures such as low temperatures or high altitudes, in contrast to the extreme conditions experienced by CS, the synthesis of amino acids in CC and CM is subject to fewer constraints. Consequently, this leads to higher protein and amino acid contents in CC and CM compared to CS.

Subsequently, we performed proteomics analysis to investigate the differences in protein expression among CS, CC, and CM. Through GO functional annotation, we found that the total proteins of the three species were predominantly enriched in metabolic processes and cellular processes, indicating that these proteins are highly active in these biological processes. By screening and comparison, 495 common DEPs were identified, including glutamate oxalacetate transaminase 2 (GOT2), carboxypeptidase A1 (CPA1), argininosuccinate synthase 1 (ASS1), glutamate dehydrogenase (GDH), and aspartate transcarbamoylase (ATCase), which were significantly enriched in alanine, aspartate, and glutamate metabolism pathways. Compared with CS and CC, GOT2 is highly expressed in CM. As an aspartate transaminase, GOT2 catalyzes the transamination of aspartate and α-ketoglutarate to produce oxaloacetate and glutamate, playing a crucial role in amino acid metabolism by linking nitrogen assimilation with the TCA cycle [40]. Among these enzymes, CPA1, ASS1, and GDH, critical enzymes for amino acid and protein synthesis, show significantly higher expression in CC and CM compared to CS. Specifically, CPA1 catalyzes the production of carbamoyl phosphate, which serves as a substrate for the urea cycle and subsequent arginine biosynthesis [41], and ASS1 further catalyzes the condensation of citrulline with aspartic acid to form argininosuccinate, thereby facilitating both the urea cycle and arginine biosynthesis [40]. In nitrogen metabolism, GDH regulates glutamate and α-ketoglutarate levels and indirectly supports amino acid biosynthesis [42], thereby contributing to carbon and nitrogen homeostasis. Additionally, GDH facilitates the amination of α-ketoglutarate in the presence of ammonia to synthesize glutamate [43]. These enzymes collectively regulate nitrogen metabolism, amino acid biosynthesis, and nucleotide production, thereby directly or indirectly supporting protein synthesis. Consequently, the upregulation of these key enzymes increases the intracellular aspartate levels and enhances the availability of upstream amino acid precursors. Furthermore, our study has revealed that these DEPs may hold potential pharmacological significance. Previous findings demonstrate that upon the knockout of GOT2 using CRISPR/Cas9 technology, pancreatic cancer cells deficient in GOT2 exhibited markedly restricted growth in immunocompetent syngeneic mouse models. These results suggest that GOT2 modulates tumor progression via regulation of the immune response within the tumor microenvironment, potentially identifying it as a novel therapeutic target [44]. Moreover, as CPA1 is a key enzyme implicated in protein digestion and metabolism, it has been shown to be closely associated with chronic pancreatitis [45]. Additionally, carboxypeptidase expression is significantly downregulated in pancreatic ductal adenocarcinoma (PDAC) tumor tissues, suggesting its potential as a novel biomarker for PDAC patients [46].

GO enrichment revealed that DEPs significantly enriched in lipid metabolic processes were involved in regulating the degradation of valine, leucine, and isoleucine. We observed a high abundance of highly expressed transaminases and oxidoreductases in CM and CC. Among these enzymes, transaminase EC: 2.6.1.42 (BCAT) played a pivotal role in this pathway. It was significantly upregulated in CM and CC, catalyzing the transfer of amino groups from L-valine, L-leucine, and L-isoleucine to α-ketoglutarate, thereby generating their corresponding α-keto acids (e.g., 3-methyl-2-oxobutanoate) and glutamate. This process laid the foundation for subsequent transamination reactions that form other amino acids. Furthermore, we found that EC: 1.2.4.4 (BCKDHA) and EC: 1.3.8.5 (ACADSB) exhibited consistent expression trends, with significant upregulation in CM and CC and relatively decreased expression in CS. These differences in enzyme expression were attributed to variations in intermediate products, leading to distinct changes in downstream metabolic reactions. Previous studies have demonstrated that EC: 2.3.1.9 (ACAT) participates in the synthesis of acyl-CoA, generating the corresponding acyl-CoA derivatives that serve as essential intermediates for the degradation of BCAAs [47]. Notably, this pathway is closely interconnected with the tricarboxylic acid (TCA) cycle. Acetyl-CoA not only serves as a precursor for branched-chain amino acid synthesis but also plays a critical role in the TCA cycle [48], highlighting the significant differences in substance and energy exchange among different *Cordyceps* species in maintaining organismal quality and function. Furthermore, in both CC and CM, the high expression of BCKDHA not only serves as an energy source for cells but also suggests that the BCAA metabolic pathway may offer novel therapeutic targets for pancreatic cancer [49]. Tao et al. demonstrated that the high-dose herb pair of *Salvia miltiorrhiza* and *Panax notoginseng* (DQ) activates BCKDHA while inhibiting BCKDHK. This dual action promotes the catabolism of BCAAs in the myocardium of rats with acute myocardial ischemia (AMI), thereby enhancing the therapeutic efficacy of DQ in mitigating AMI-induced injury [50]. In conclusion, the protein and amino acid contents exhibit substantial variation among CS, CC, and CM. The differential expression of enzymes associated with the biosynthesis and degradation of BCAAs plays a pivotal role in driving the amino acid metabolic pathways, which are essential for unlocking the potential functionalities of fungal proteins.

## 5. Conclusions

Overall, this study demonstrates that the protein and amino acid contents in CC and CM are higher than in CS. However, the elevated Leu, ILE, and His contents in CS may result from prolonged low-temperature, high-altitude adaptation, enhancing membrane protection and reactive oxygen species scavenging capacity. Proteomics analysis reveals that proteins associated with amino acid metabolic processes and α-amino acid biosynthetic pathways are more abundant in CC and CM than in CS. This suggests that CC and CM may possess more active mechanisms for synthesizing primary metabolite proteins and amino acids compared to CS. In comparison to CS, CC and CM exhibit relatively higher expression levels of BCKDHA and ACADSB, which play critical roles in amino acid catabolism. CPA1, ASS1, and GDH have also been identified as key regulators of protein synthesis within the alanine, aspartate, and glutamate metabolic pathways. Based on these findings, we propose that BCKDHA, CPA1, and GDH may be potential protein markers for evaluating CS, CC, and CM protein quality.

## Data Availability

The mass spectrometry proteomics data for *Cordyceps* have been deposited to the ProteomeXchange Consortium (http://proteomecentral.proteomexchange.org/cgi/GetDataset?ID=PXD063259, accessed on 23 May 2025) via the iProX partner repository with the dataset identifier PXD063259.

## Supplementary Materials

The following supporting information can be downloaded at https://www.mdpi.com/article/10.3390/jof11050365/s1: Figure S1: Standard curve for amino acid quantitative analysis; Figure S2: Quantitative analysis of non-essential amino acids in CS, CM, and CC. (A) Arginine content; (B) Proline content; (C) Tyrosine content; (D) Alanine content; (E) Serine content; (F) Glycine content; (G) Glutamic acid content; (H) Glutamine content; (I) Cysteine content; (J) Asparagine content; (K) Aspartic acid content.

## Abbreviations

The following abbreviations are used in this manuscript:

*Cordyceps sinensis*	CS
*Cordyceps militaris*	CM
*Cordyceps cicadae*	CC
Four-dimensional data-independent acquisition	4D-DIA
Differentially expressed proteins	DEPs
Essential amino acids	EAAs
Non-essential amino acids	NEAAs
Carboxypeptidase A1	CPA1
Argininosuccinate synthase 1	ASS1
Branched-chain keto acid dehydrogenase E1 subunit alpha	BCKDHA
Acyl-CoA dehydrogenase short/branched chain	ACADSB
Heat shock proteins	HSPs
Liquid chromatography	LC
Data-independent acquisition	DIA
Mass spectrometry	MS
Maximum Entropy	MaxEnt
Gene Ontology	GO
Kyoto Encyclopedia of Genes and Genomes	KEGG
Pyruvate carboxylase	PC
Fumarate hydratase	FH
Dihydrolipoyl dehydrogenase	DLD
Deoxyuridine 5′-triphosphate nucleotidohydrolase	DUT
Uracil phosphoribosyltransferase	UPRT
Principal component analysis	PCA
Branched-chain amino acids	BCAAs
Branched-chain-amino-acid aminotransferase	BCAT
Branched-chain dihydrolipoamide acyltransferase	BCDAT
Medium-chain specific acyl-CoA dehydrogenase	ACADM
3-hydroxyisobutyryl-CoA hydrolase	HIBCH
Acetyl-CoA acetyltransferase	ACAT
Glutamate oxalacetate transaminases 2	GOT2
Glutamate dehydrogenase	GDH
Aspartate transcarbamoylase	ATCase
Pancreatic ductal adenocarcinoma	PDAC
Acute myocardial ischemia	AMI
