# Supplementary material for: Integrated Amino Acid Profiling and 4D-DIA Proteomics Reveal Protein Quality Divergence and Metabolic Adaptation in Cordyceps Species
Source: J Fungi (Basel). 2025 May 8;11(5):365. doi: 10.3390/jof11050365 (PMC12113033; doi:10.3390/jof11050365)

Analyte Name: Ala_1
Internal Standard: *No data for IS Peak Name*

**Data File**	2024 12 06 STD AA.wiff	**Result Table**	RW2024112111_AA_ZDD_2024 12 12.rdb
**Acquisition Date**	12/6/2024 7:47:21 PM	**Algorithm Used**	MQL
**Acquisition Method**	Amino acid_Test_YJX_20240827.dam	**Instrument Name**	AB SCIEX QTRAP 5500
**Project**	Products		

Regression Equation: y = -9.99e+003 x^2 + 1.87e+006 x + 4.36e+003 (r = 0.9998)

Expected Concentration	Number of Values	Mean Calculated Concentration	% Accuracy	Std. Deviation	%CV
0.01	1	0.01	93.3	NaN	NaN
0.05	1	0.05	106.7	NaN	NaN
0.1	1	0.11	106.2	NaN	NaN
1	1	0.96	96.3	NaN	NaN
5	1	4.83	96.6	NaN	NaN
10	1	9.85	98.5	NaN	NaN
20	1	20.60	103.0	NaN	NaN
50	1	49.69	99.4	NaN	NaN

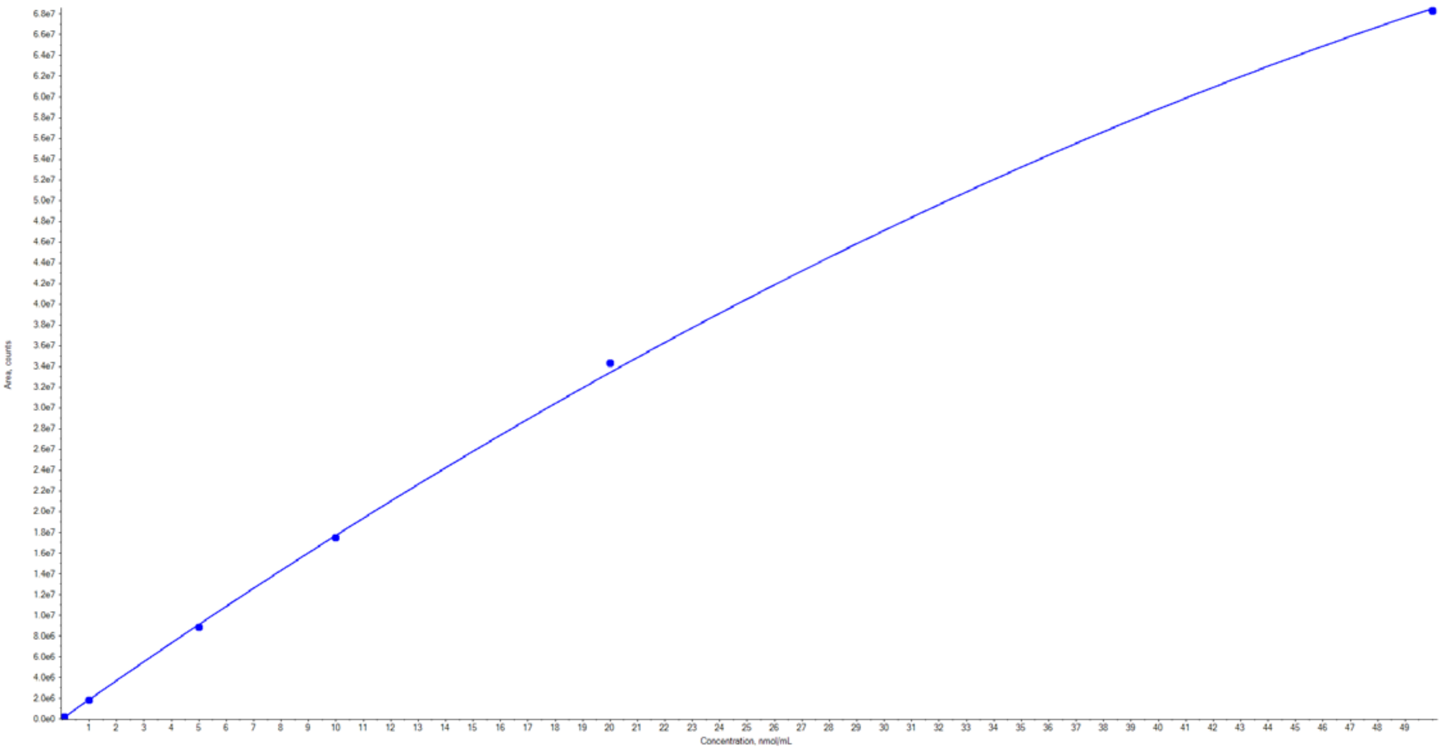

Analyte Name: Val_2
Internal Standard: *No data for IS Peak Name*

**Data File**	2024 12 06 STD AA.wiff	**Result Table**	RW2024112111_AA_ZDD_2024 12 12.rdb
**Acquisition Date**	12/6/2024 7:47:21 PM	**Algorithm Used**	MQL
**Acquisition Method**	Amino acid_Test_YJX_20240827.dam	**Instrument Name**	AB SCIEX QTRAP 5500
**Project**	Products		

Regression Equation: y = -2.36e+004 x^2 + 3.05e+006 x + 1.93e+004 (r = 1.0000)

Expected Concentration	Number of Values	Mean Calculated Concentration	% Accuracy	Std. Deviation	%CV
0.01	1	0.01	108.1	NaN	NaN
0.05	1	0.05	93.3	NaN	NaN
0.1	1	0.10	100.2	NaN	NaN
1	1	0.98	97.7	NaN	NaN
5	1	5.08	101.7	NaN	NaN
10	1	9.83	98.3	NaN	NaN
20	1	20.16	100.8	NaN	NaN
50	1	49.90	99.8	NaN	NaN

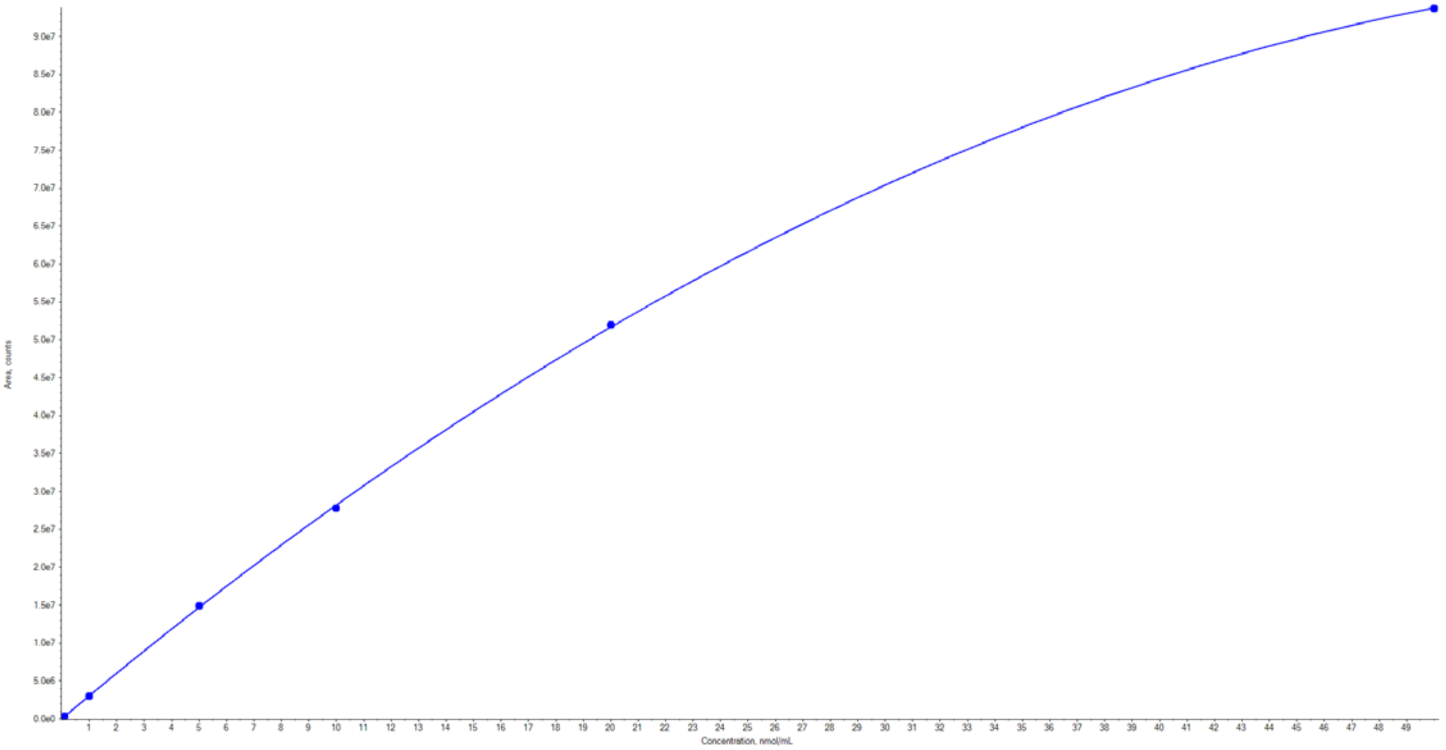

Analyte Name: Leu_2
Internal Standard: *No data for IS Peak Name*

**Data File**	2024 12 06 STD AA.wiff	**Result Table**	RW2024112111_AA_ZDD_2024 12 12.rdb
**Acquisition Date**	12/6/2024 7:47:21 PM	**Algorithm Used**	MQL
**Acquisition Method**	Amino acid_Test_YJX_20240827.dam	**Instrument Name**	AB SCIEX QTRAP 5500
**Project**	Products		

Regression Equation: y = -127 x^2 + 2.59e+004 x + 611 (r = 0.9996)

Expected Concentration	Number of Values	Mean Calculated Concentration	% Accuracy	Std. Deviation	%CV
0.01	1	0.01	86.9	NaN	NaN
0.05	1	0.05	101.7	NaN	NaN
0.1	1	0.10	103.0	NaN	NaN
1	1	1.12	112.2	NaN	NaN
5	1	4.93	98.6	NaN	NaN
10	1	9.48	94.8	NaN	NaN
20	1	20.66	103.3	NaN	NaN
50	1	49.78	99.6	NaN	NaN

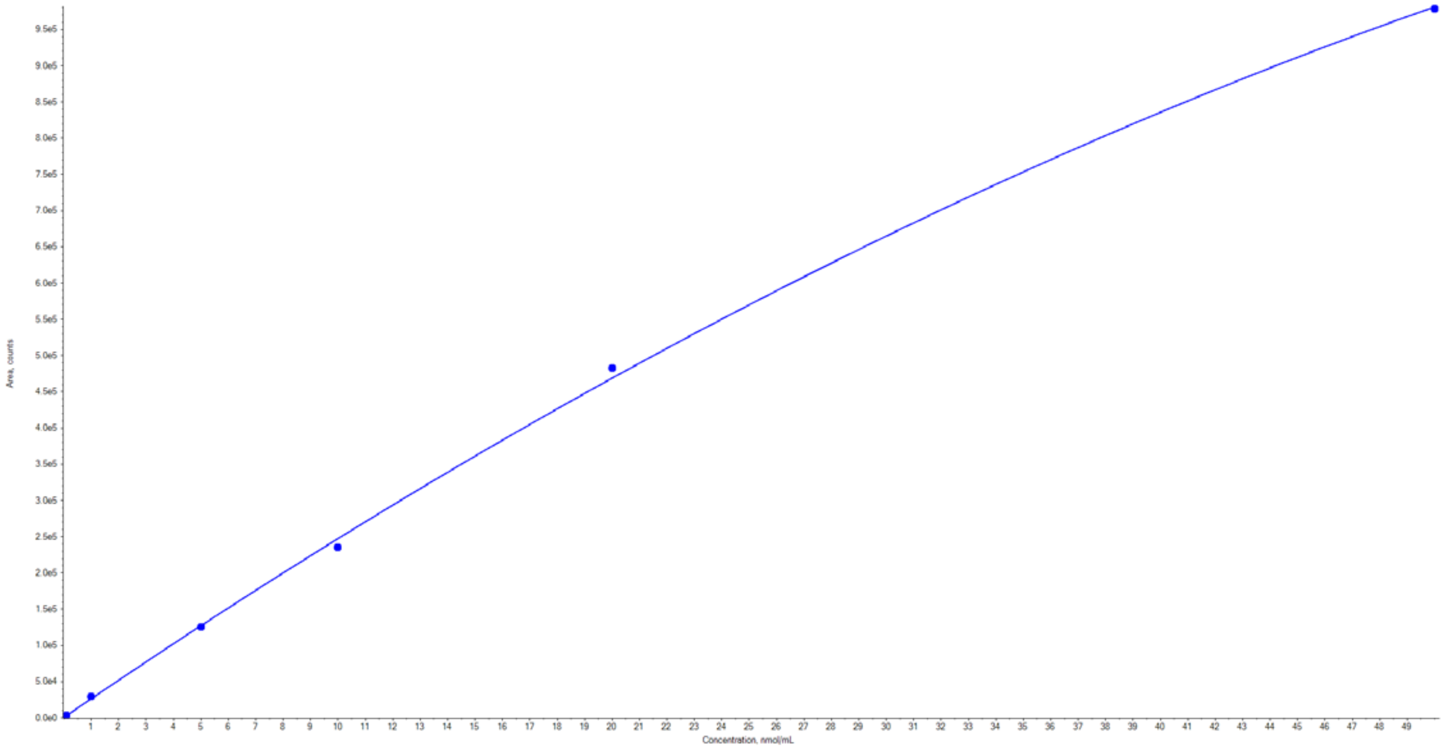

Analyte Name: Ile_2
Internal Standard: *No data for IS Peak Name*

**Data File**	2024 12 06 STD AA.wiff	**Result Table**	RW2024112111_AA_ZDD_2024 12 12.rdb
**Acquisition Date**	12/6/2024 7:47:21 PM	**Algorithm Used**	MQL
**Acquisition Method**	Amino acid_Test_YJX_20240827.dam	**Instrument Name**	AB SCIEX QTRAP 5500
**Project**	Products		

Regression Equation: y = -1.49e+004 x^2 + 2.05e+006 x + 3.44e+003 (r = 1.0000)

Expected Concentration	Number of Values	Mean Calculated Concentration	% Accuracy	Std. Deviation	%CV
0.01	1	0.01	100.6	NaN	NaN
0.05	1	0.05	100.7	NaN	NaN
0.1	1	0.10	101.0	NaN	NaN
1	1	0.97	96.6	NaN	NaN
5	1	5.03	100.7	NaN	NaN
10	1	10.16	101.6	NaN	NaN
20	1	19.75	98.8	NaN	NaN
50	1	50.15	100.3	NaN	NaN

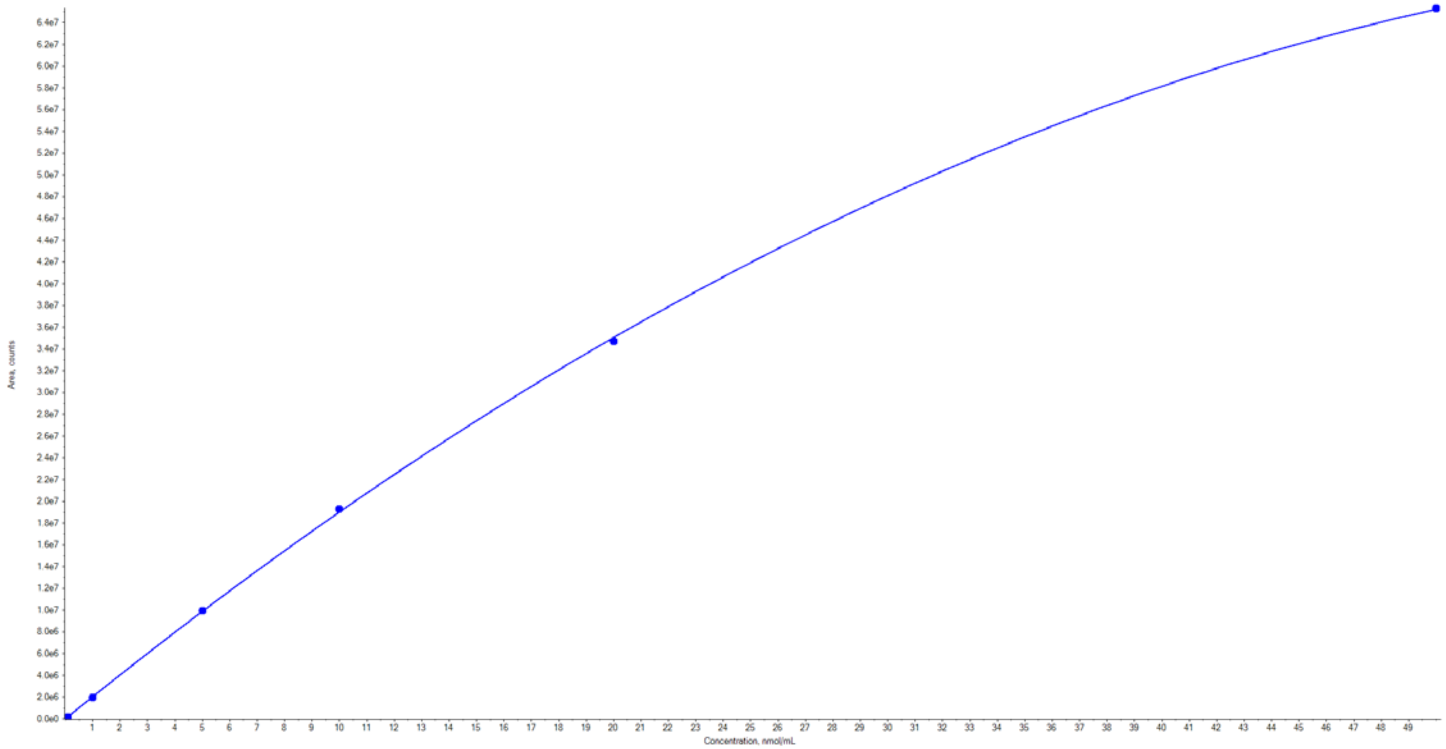

Analyte Name: Glu_1
Internal Standard: *No data for IS Peak Name*

**Data File**	2024 12 06 STD AA.wiff	**Result Table**	RW2024112111_AA_ZDD_2024 12 12.rdb
**Acquisition Date**	12/6/2024 7:47:21 PM	**Algorithm Used**	MQL
**Acquisition Method**	Amino acid_Test_YJX_20240827.dam	**Instrument Name**	AB SCIEX QTRAP 5500
**Project**	Products		

Regression Equation: y = 2.55e+006 x (r = 0.9998)

Expected Concentration	Number of Values	Mean Calculated Concentration	% Accuracy	Std. Deviation	%CV
0.05	1	0.05	102.1	NaN	NaN
0.1	1	0.11	106.2	NaN	NaN
1	1	0.97	96.6	NaN	NaN
5	1	4.89	97.8	NaN	NaN
10	1	10.05	100.5	NaN	NaN
20	1	20.64	103.2	NaN	NaN
50	1	49.44	98.9	NaN	NaN

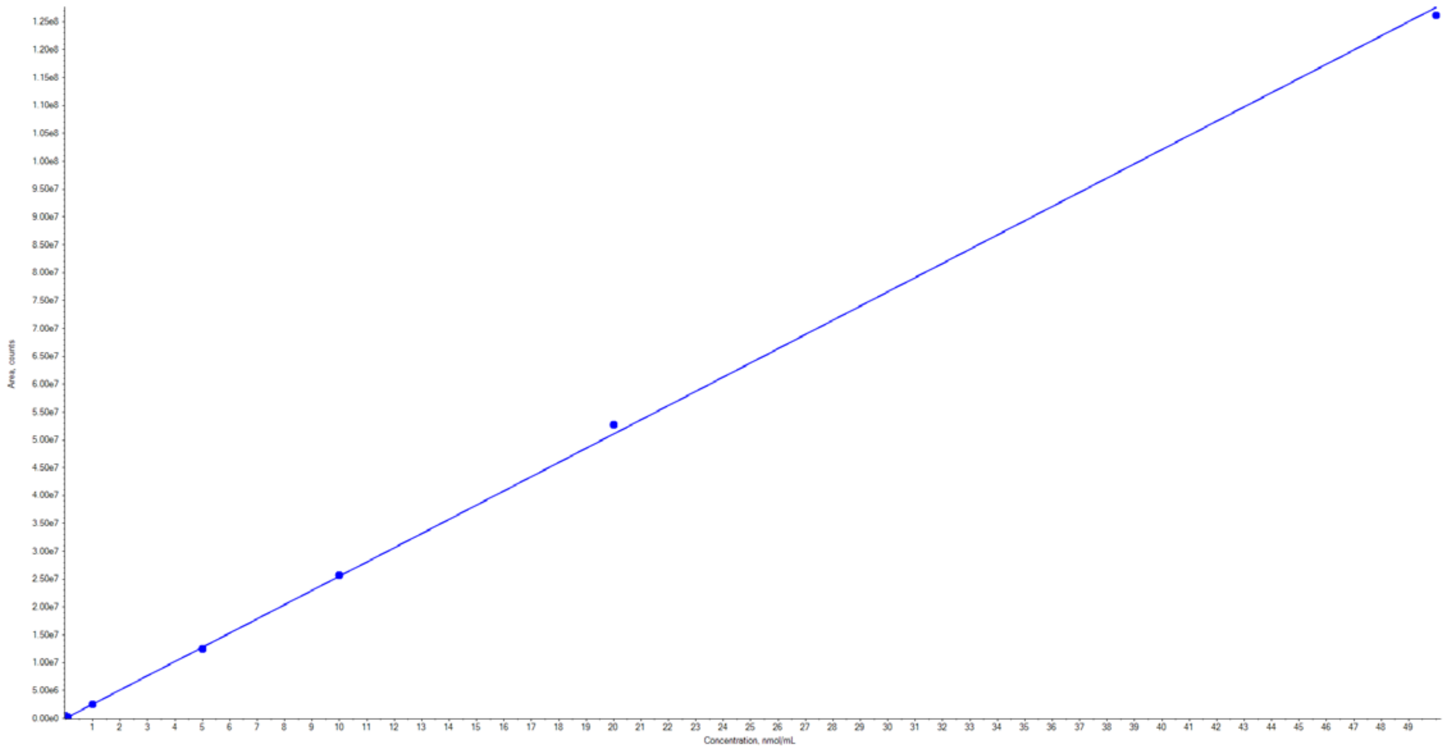

Analyte Name: Lys_1
Internal Standard: *No data for IS Peak Name*

**Data File**	2024 12 06 STD AA.wiff	**Result Table**	RW2024112111_AA_ZDD_2024 12 12.rdb
**Acquisition Date**	12/6/2024 7:47:21 PM	**Algorithm Used**	MQL
**Acquisition Method**	Amino acid_Test_YJX_20240827.dam	**Instrument Name**	AB SCIEX QTRAP 5500
**Project**	Products		

Regression Equation: y = 2.17e+006 x + -2.44e+004 (r = 0.9958)

Expected Concentration	Number of Values	Mean Calculated Concentration	% Accuracy	Std. Deviation	%CV
0.01	1	0.02	240.4	NaN	NaN
0.05	1	0.03	52.0	NaN	NaN
0.1	1	0.05	53.3	NaN	NaN
1	1	0.56	55.7	NaN	NaN
5	1	4.41	88.2	NaN	NaN
10	1	10.02	100.2	NaN	NaN
20	1	22.64	113.2	NaN	NaN
50	1	48.43	96.9	NaN	NaN

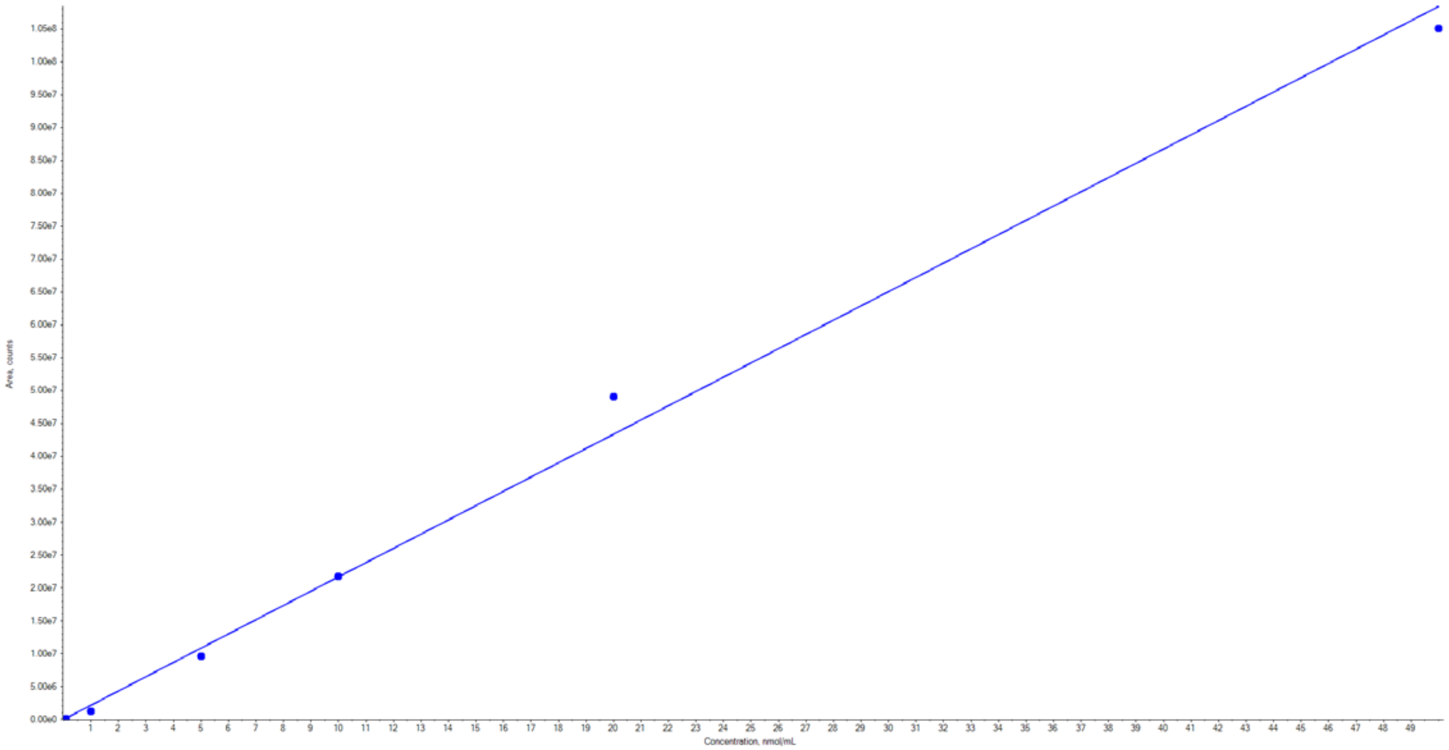

Analyte Name: Arg_2
Internal Standard: *No data for IS Peak Name*

**Data File**	2024 12 06 STD AA.wiff	**Result Table**	RW2024112111_AA_ZDD_2024 12 12.rdb
**Acquisition Date**	12/6/2024 7:47:21 PM	**Algorithm Used**	MQL
**Acquisition Method**	Amino acid_Test_YJX_20240827.dam	**Instrument Name**	AB SCIEX QTRAP 5500
**Project**	Products		

Regression Equation: y = 7.26e+005 x + 3.95e+003 (r = 0.9979)

Expected Concentration	Number of Values	Mean Calculated Concentration	% Accuracy	Std. Deviation	%CV
0.01	1	0.02	198.7	NaN	NaN
0.05	1	0.03	68.6	NaN	NaN
0.1	1	0.07	69.4	NaN	NaN
1	1	0.73	73.0	NaN	NaN
5	1	4.36	87.3	NaN	NaN
10	1	9.51	95.1	NaN	NaN
20	1	21.73	108.7	NaN	NaN
50	1	49.70	99.4	NaN	NaN

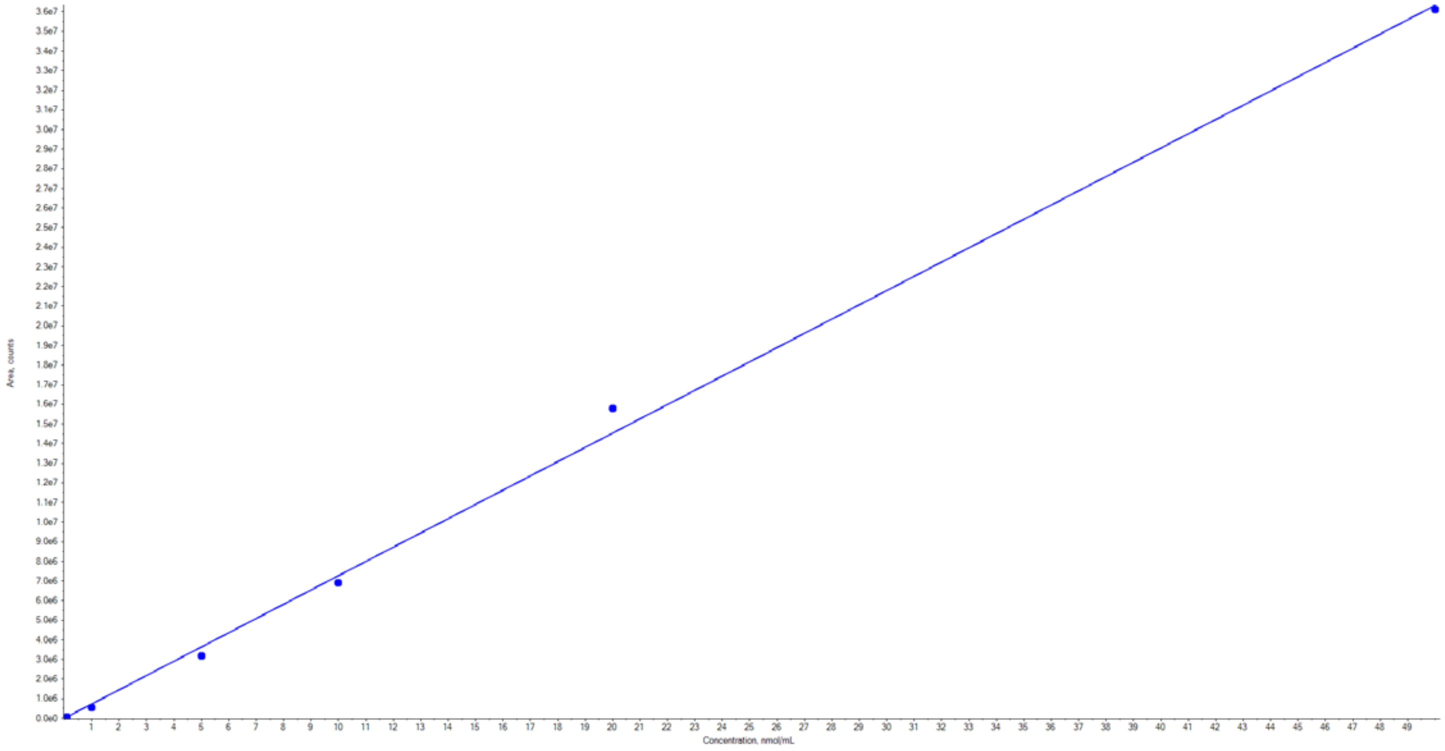

Analyte Name: Gly_1
Internal Standard: *No data for IS Peak Name*

**Data File**	2024 12 06 STD AA.wiff	**Result Table**	RW2024112111_AA_ZDD_2024 12 12.rdb
**Acquisition Date**	12/6/2024 7:47:21 PM	**Algorithm Used**	MQL
**Acquisition Method**	Amino acid_Test_YJX_20240827.dam	**Instrument Name**	AB SCIEX QTRAP 5500
**Project**	Products		

Regression Equation: y = 4.84e+004 x + 1.68e+003 (r = 0.9988)

Expected Concentration	Number of Values	Mean Calculated Concentration	% Accuracy	Std. Deviation	%CV
0.01	1	0.01	93.0	NaN	NaN
0.05	1	0.05	100.3	NaN	NaN
0.1	1	0.09	89.0	NaN	NaN
1	1	1.05	105.2	NaN	NaN
5	1	5.20	103.9	NaN	NaN
10	1	10.75	107.5	NaN	NaN
20	1	21.03	105.1	NaN	NaN
50	1	47.99	96.0	NaN	NaN

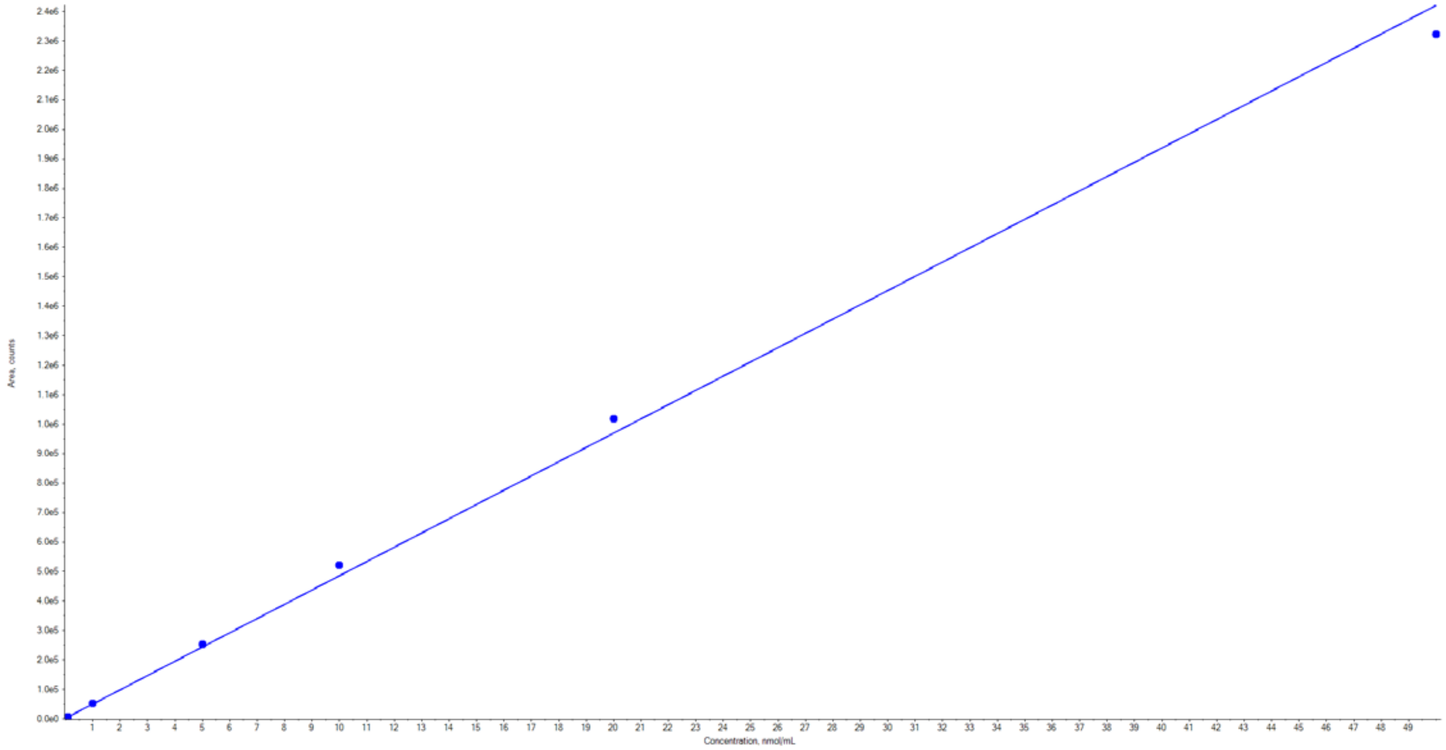

Analyte Name: Ser_1
Internal Standard: *No data for IS Peak Name*

**Data File**	2024 12 06 STD AA.wiff	**Result Table**	RW2024112111_AA_ZDD_2024 12 12.rdb
**Acquisition Date**	12/6/2024 7:47:21 PM	**Algorithm Used**	MQL
**Acquisition Method**	Amino acid_Test_YJX_20240827.dam	**Instrument Name**	AB SCIEX QTRAP 5500
**Project**	Products		

Regression Equation: y = -1.25e+004 x^2 + 2.16e+006 x + 4.26e+004 (r = 0.9998)

Expected Concentration	Number of Values	Mean Calculated Concentration	% Accuracy	Std. Deviation	%CV
0.05	1	0.05	104.9	NaN	NaN
0.1	1	0.10	99.2	NaN	NaN
1	1	0.99	99.3	NaN	NaN
5	1	4.77	95.4	NaN	NaN
10	1	9.86	98.6	NaN	NaN
20	1	20.65	103.3	NaN	NaN
50	1	49.64	99.3	NaN	NaN

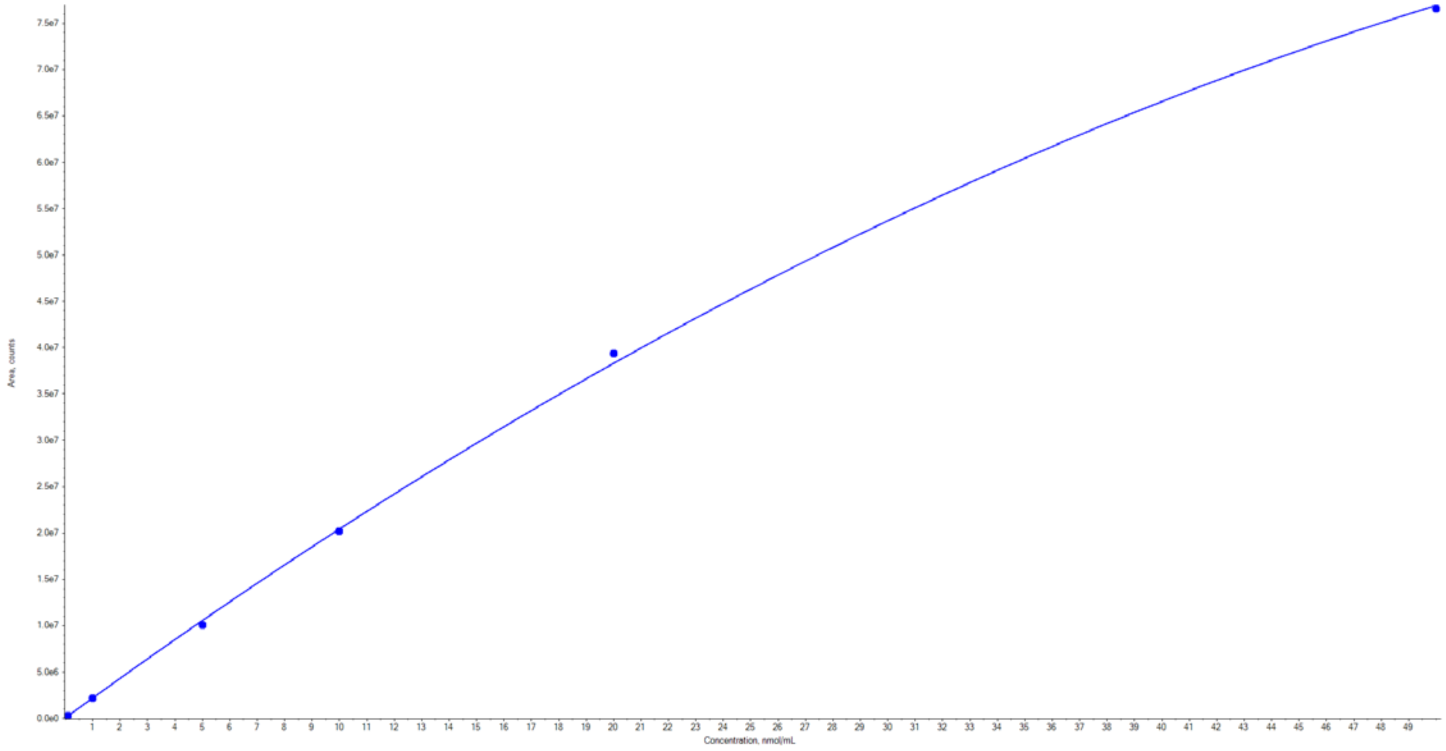

Analyte Name: Thr_1
Internal Standard: *No data for IS Peak Name*

**Data File**	2024 12 06 STD AA.wiff	**Result Table**	RW2024112111_AA_ZDD_2024 12 12.rdb
**Acquisition Date**	12/6/2024 7:47:21 PM	**Algorithm Used**	MQL
**Acquisition Method**	Amino acid_Test_YJX_20240827.dam	**Instrument Name**	AB SCIEX QTRAP 5500
**Project**	Products		

Regression Equation: y = 1.43e+006 x (r = 0.9959)

Expected Concentration	Number of Values	Mean Calculated Concentration	% Accuracy	Std. Deviation	%CV
0.05	1	0.05	108.3	NaN	NaN
0.1	1	0.12	117.9	NaN	NaN
1	1	1.15	115.5	NaN	NaN
5	1	5.48	109.6	NaN	NaN
10	1	11.39	113.9	NaN	NaN
20	1	21.74	108.7	NaN	NaN
50	1	46.22	92.4	NaN	NaN

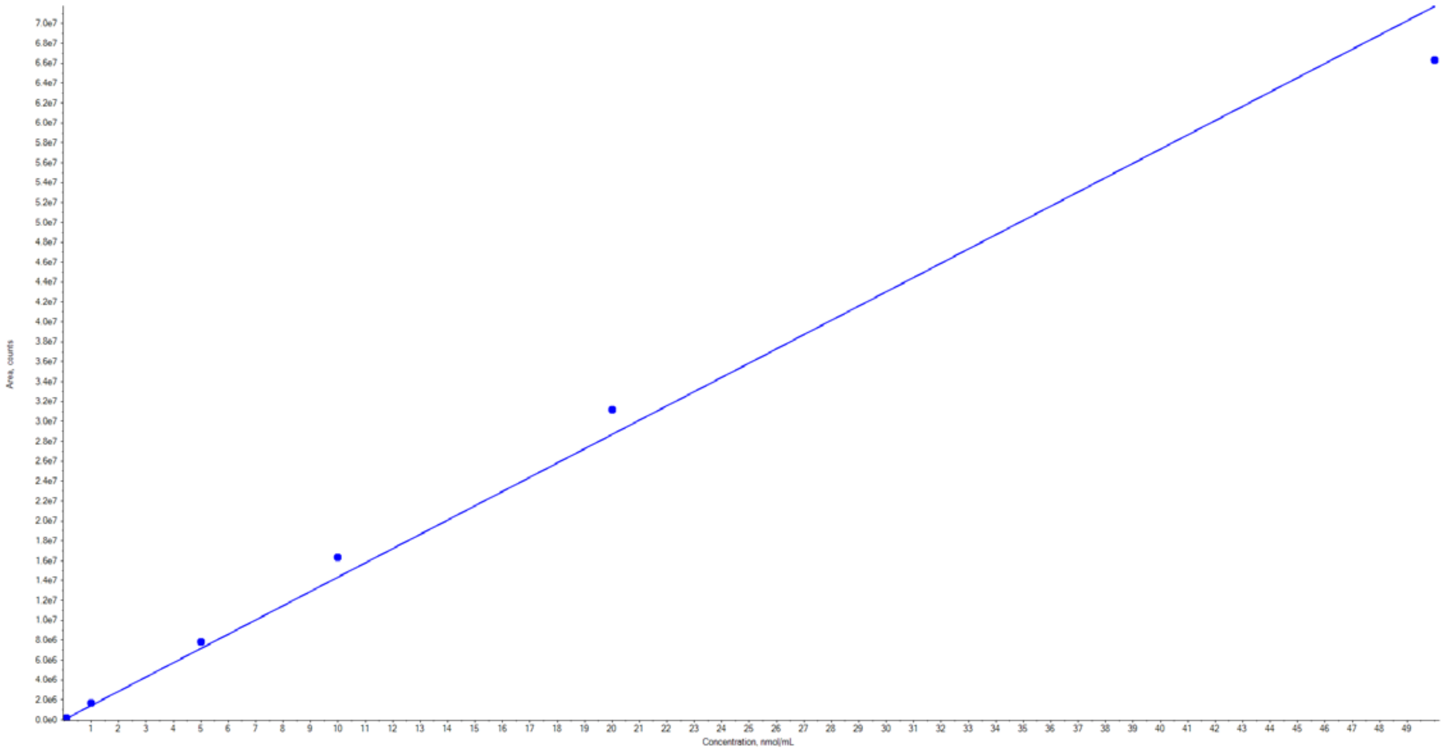

Analyte Name: Cys_1
Internal Standard: *No data for IS Peak Name*

**Data File**	2024 12 06 STD AA.wiff	**Result Table**	RW2024112111_AA_ZDD_2024 12 12.rdb
**Acquisition Date**	12/6/2024 7:47:21 PM	**Algorithm Used**	MQL
**Acquisition Method**	Amino acid_Test_YJX_20240827.dam	**Instrument Name**	AB SCIEX QTRAP 5500
**Project**	Products		

Regression Equation: y = 2.79e+005 x (r = 0.9979)

Expected Concentration	Number of Values	Mean Calculated Concentration	% Accuracy	Std. Deviation	%CV
1	1	0.08	8.4	NaN	NaN
5	1	3.16	63.2	NaN	NaN
10	1	7.65	76.5	NaN	NaN
20	1	18.75	93.7	NaN	NaN
50	1	51.17	102.3	NaN	NaN

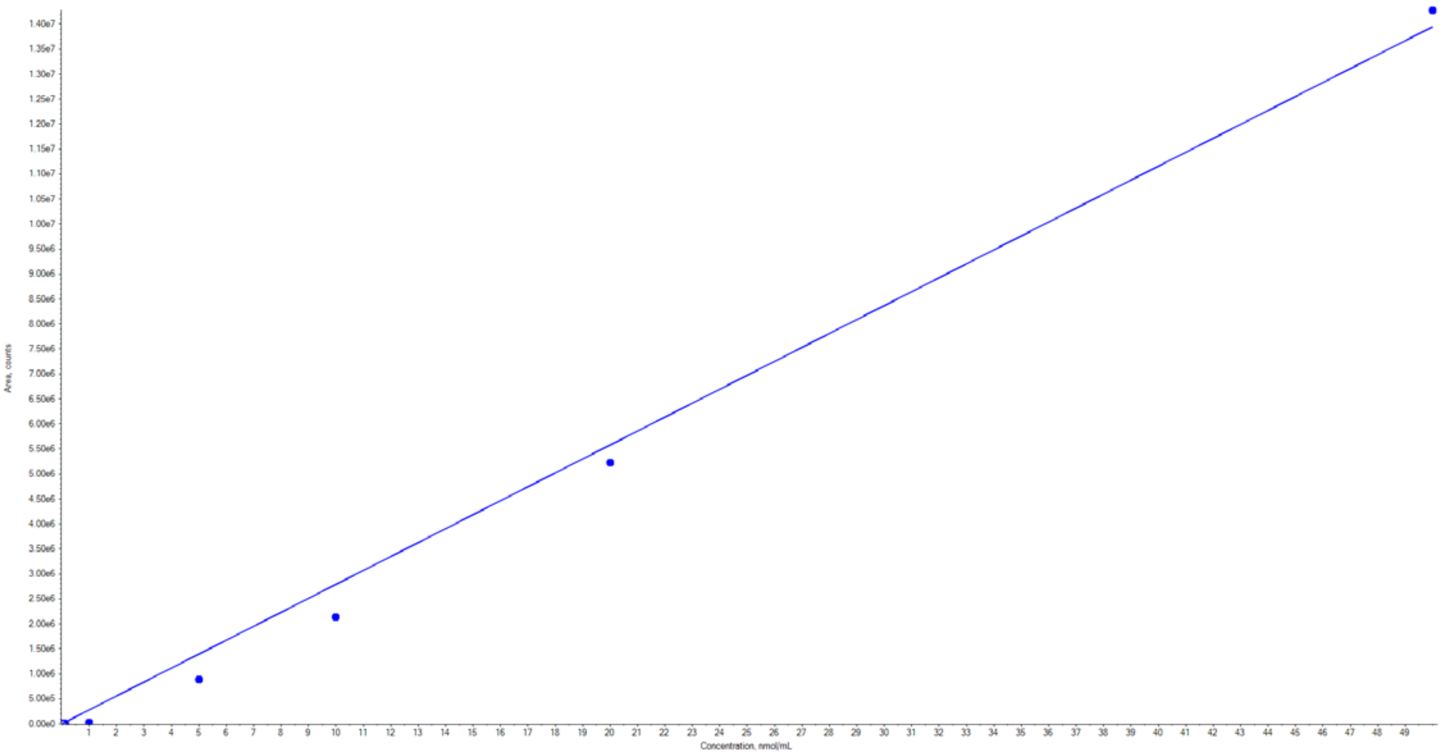

Analyte Name: Asp_1
Internal Standard: *No data for IS Peak Name*

**Data File**	2024 12 06 STD AA.wiff	**Result Table**	RW2024112111_AA_ZDD_2024 12 12.rdb
**Acquisition Date**	12/6/2024 7:47:21 PM	**Algorithm Used**	MQL
**Acquisition Method**	Amino acid_Test_YJX_20240827.dam	**Instrument Name**	AB SCIEX QTRAP 5500
**Project**	Products		

Regression Equation: y = 6.76e+005 x (r = 0.9999)

Expected Concentration	Number of Values	Mean Calculated Concentration	% Accuracy	Std. Deviation	%CV
0.01	1	0.01	101.9	NaN	NaN
0.05	1	0.05	103.9	NaN	NaN
0.1	1	0.10	103.6	NaN	NaN
1	1	1.04	103.9	NaN	NaN
5	1	5.11	102.1	NaN	NaN
10	1	10.29	102.9	NaN	NaN
20	1	20.24	101.2	NaN	NaN
50	1	49.32	98.6	NaN	NaN

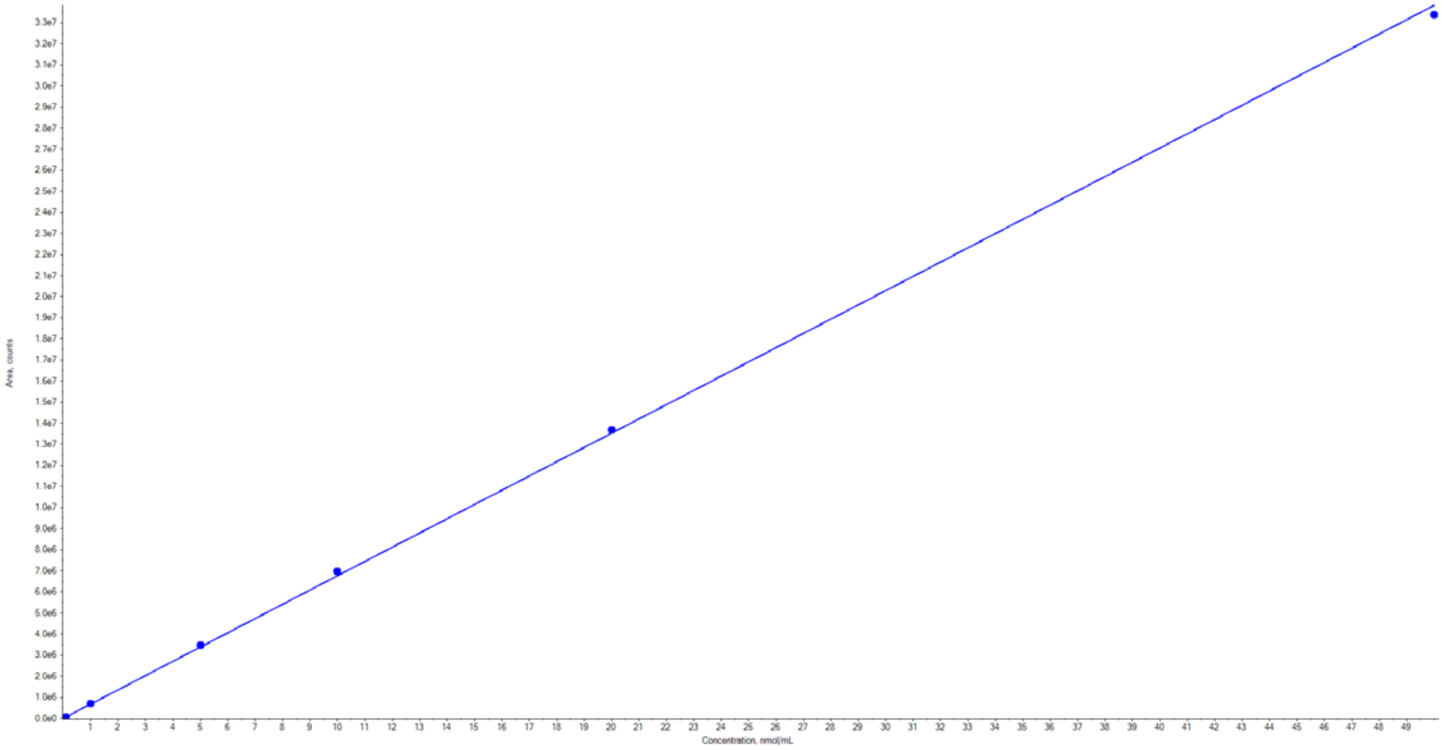

Analyte Name: Asn_1
Internal Standard: *No data for IS Peak Name*

**Data File**	2024 12 06 STD AA.wiff	**Result Table**	RW2024112111_AA_ZDD_2024 12 12.rdb
**Acquisition Date**	12/6/2024 7:47:21 PM	**Algorithm Used**	MQL
**Acquisition Method**	Amino acid_Test_YJX_20240827.dam	**Instrument Name**	AB SCIEX QTRAP 5500
**Project**	Products		

Regression Equation: y = 7.59e+005 x (r = 0.9995)

Expected Concentration	Number of Values	Mean Calculated Concentration	% Accuracy	Std. Deviation	%CV
0.01	1	0.01	136.3	NaN	NaN
0.05	1	0.05	101.9	NaN	NaN
0.1	1	0.10	100.6	NaN	NaN
1	1	1.06	105.6	NaN	NaN
5	1	5.42	108.3	NaN	NaN
10	1	10.09	100.9	NaN	NaN
20	1	20.56	102.8	NaN	NaN
50	1	48.88	97.8	NaN	NaN

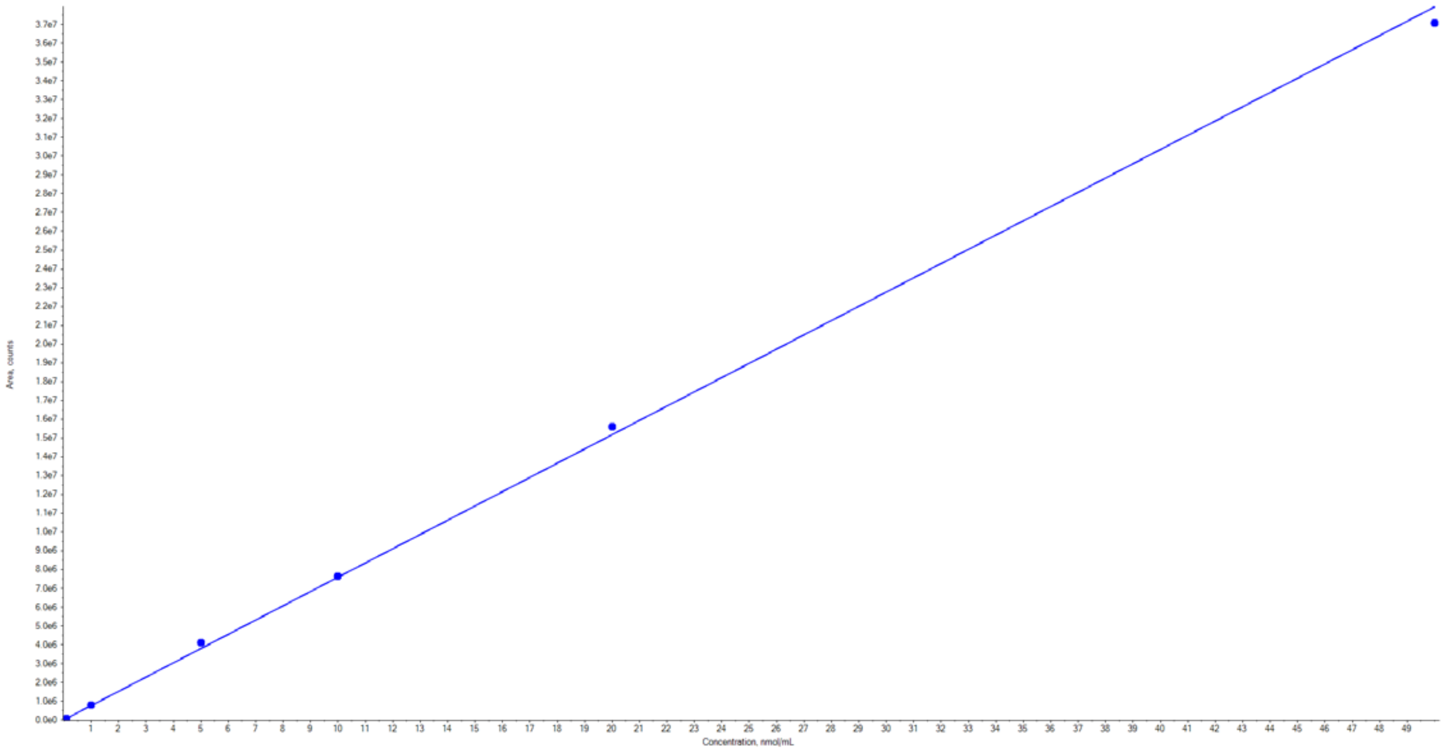

Analyte Name: Gln_1
Internal Standard: *No data for IS Peak Name*

**Data File**	2024 12 06 STD AA.wiff	**Result Table**	RW2024112111_AA_ZDD_2024 12 12.rdb
**Acquisition Date**	12/6/2024 7:47:21 PM	**Algorithm Used**	MQL
**Acquisition Method**	Amino acid_Test_YJX_20240827.dam	**Instrument Name**	AB SCIEX QTRAP 5500
**Project**	Products		

Regression Equation: y = 3.19e+005 x (r = 0.9998)

Expected Concentration	Number of Values	Mean Calculated Concentration	% Accuracy	Std. Deviation	%CV
0.05	1	0.06	110.8	NaN	NaN
0.1	1	0.10	101.2	NaN	NaN
1	1	0.94	94.1	NaN	NaN
5	1	5.30	106.0	NaN	NaN
10	1	10.20	102.0	NaN	NaN
20	1	20.32	101.6	NaN	NaN
50	1	49.23	98.5	NaN	NaN

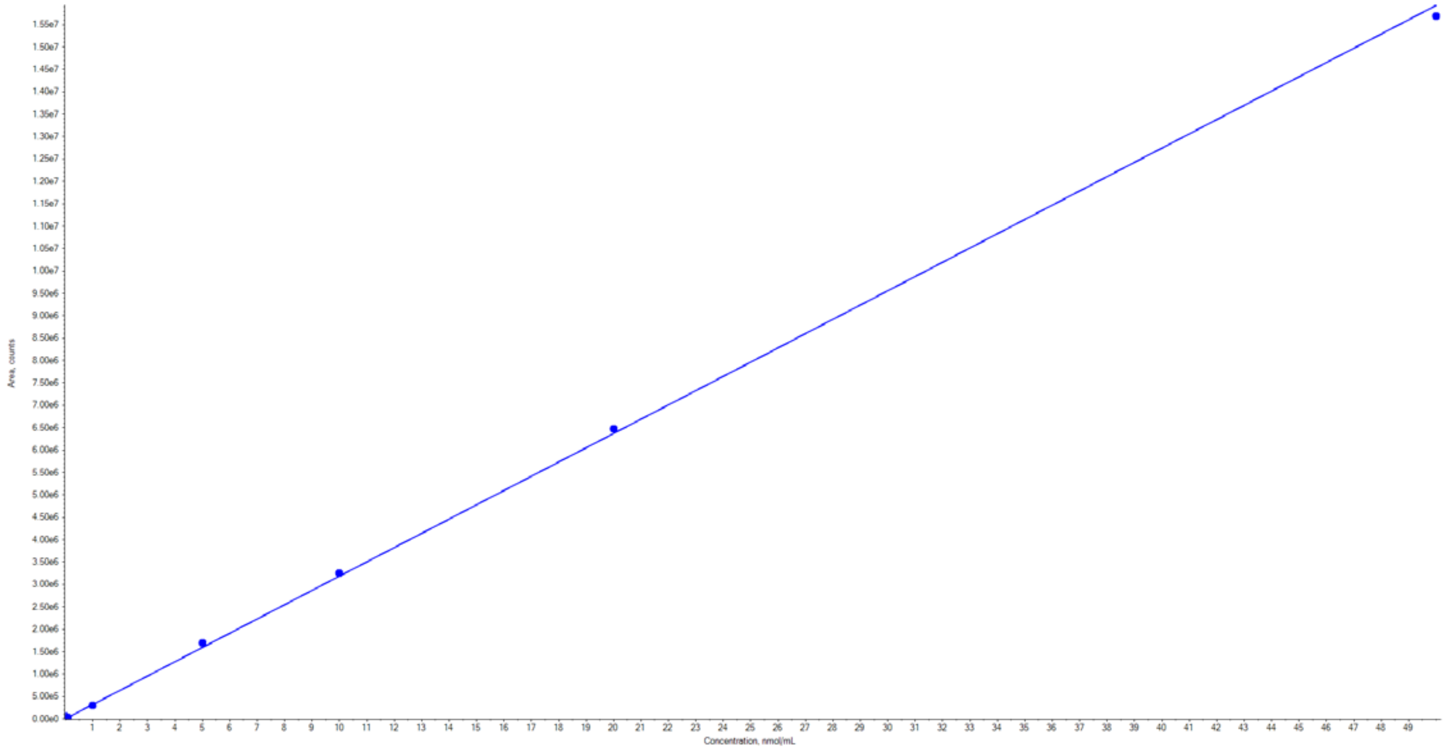

Analyte Name: Phe_2
Internal Standard: *No data for IS Peak Name*

**Data File**	2024 12 06 STD AA.wiff	**Result Table**	RW2024112111_AA_ZDD_2024 12 12.rdb
**Acquisition Date**	12/6/2024 7:47:21 PM	**Algorithm Used**	MQL
**Acquisition Method**	Amino acid_Test_YJX_20240827.dam	**Instrument Name**	AB SCIEX QTRAP 5500
**Project**	Products		

Regression Equation: y = 7.5e+006 x (r = 0.9954)

Expected Concentration	Number of Values	Mean Calculated Concentration	% Accuracy	Std. Deviation	%CV
0.01	1	0.02	190.3	NaN	NaN
0.05	1	0.06	129.9	NaN	NaN
0.1	1	0.12	123.4	NaN	NaN
1	1	1.21	121.4	NaN	NaN
5	1	5.43	108.6	NaN	NaN
10	1	9.31	93.1	NaN	NaN

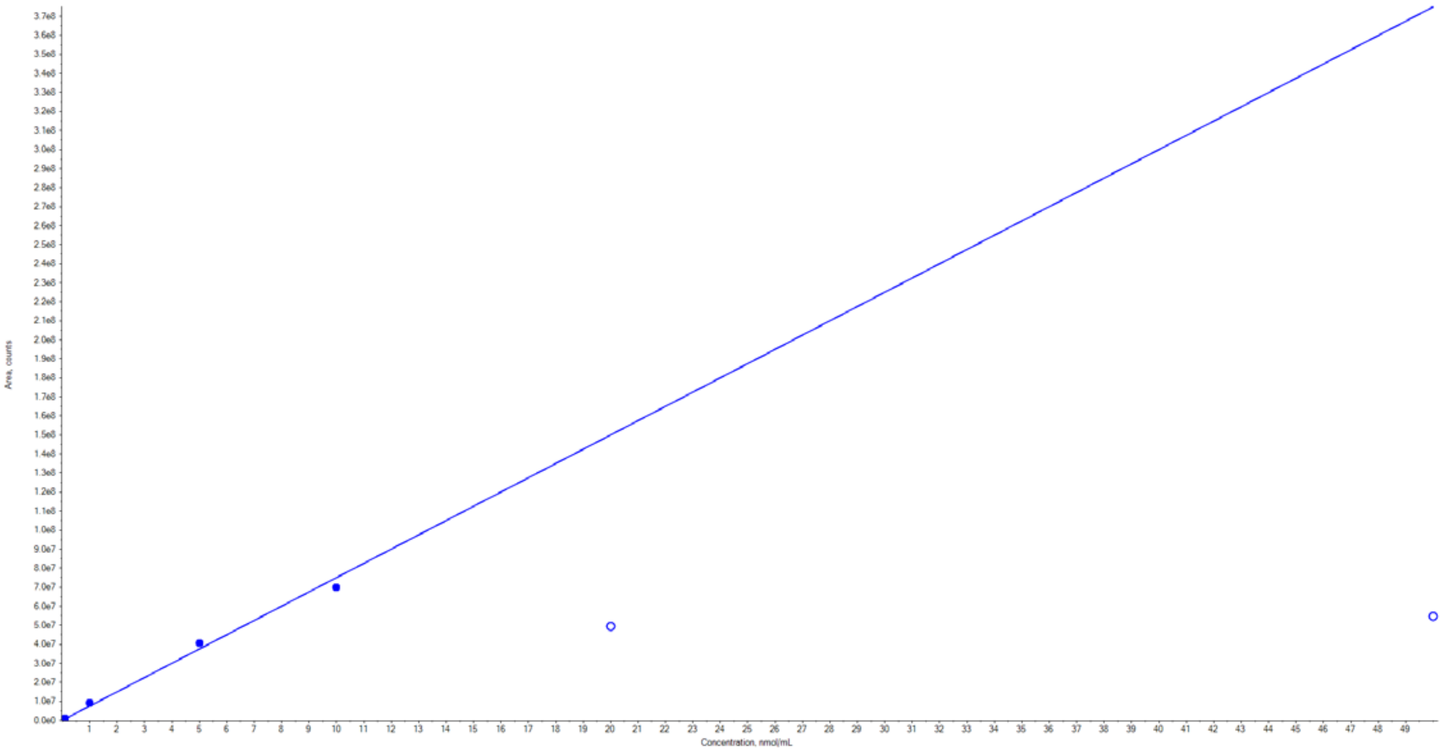

Analyte Name: Tyr_2
Internal Standard: *No data for IS Peak Name*

**Data File**	2024 12 06 STD AA.wiff	**Result Table**	RW2024112111_AA_ZDD_2024 12 12.rdb
**Acquisition Date**	12/6/2024 7:47:21 PM	**Algorithm Used**	MQL
**Acquisition Method**	Amino acid_Test_YJX_20240827.dam	**Instrument Name**	AB SCIEX QTRAP 5500
**Project**	Products		

Regression Equation: y = 8.38e+005 x (r = 0.9977)

Expected Concentration	Number of Values	Mean Calculated Concentration	% Accuracy	Std. Deviation	%CV
0.01	1	0.02	205.2	NaN	NaN
0.05	1	0.07	134.5	NaN	NaN
0.1	1	0.13	125.5	NaN	NaN
1	1	1.12	112.4	NaN	NaN
5	1	5.52	110.4	NaN	NaN
10	1	10.71	107.1	NaN	NaN
20	1	21.40	107.0	NaN	NaN
50	1	47.19	94.4	NaN	NaN

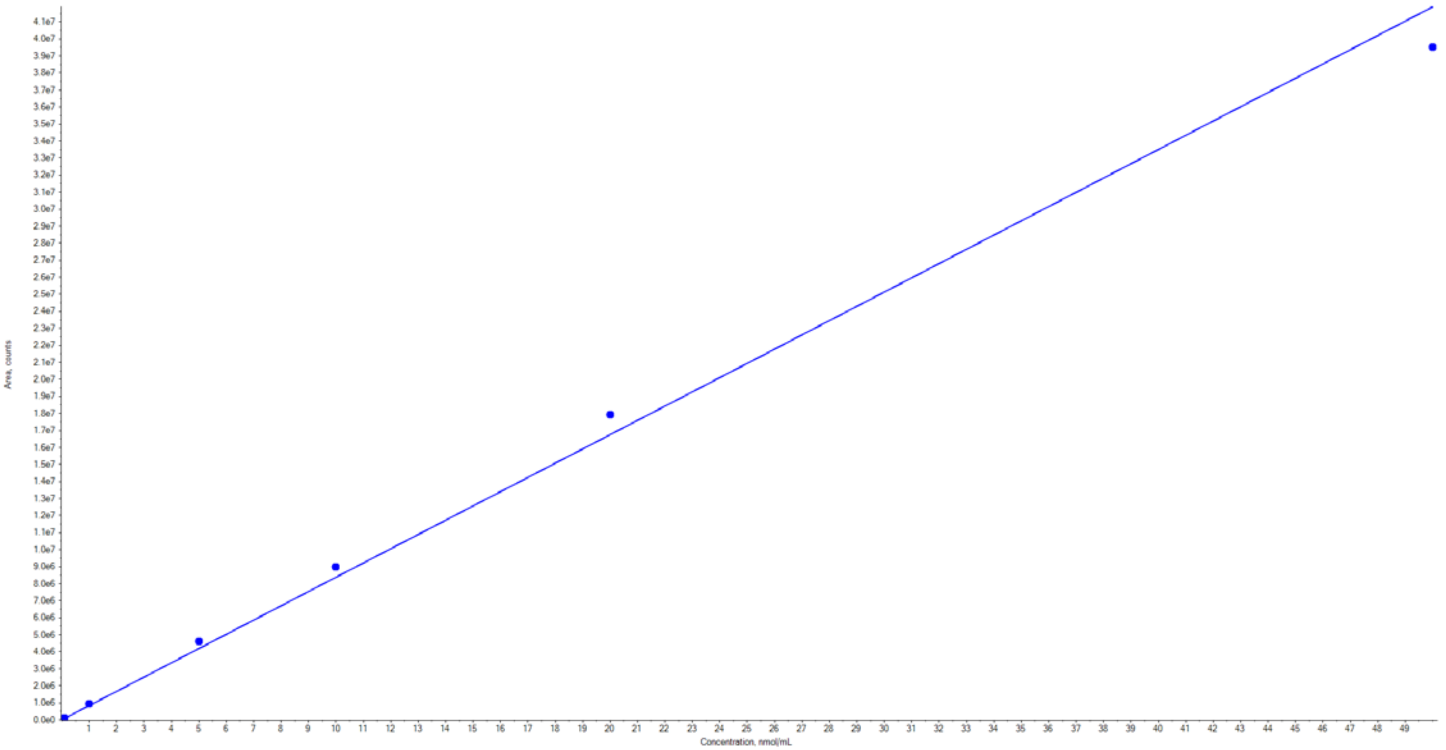

Analyte Name: Trp_1
Internal Standard: *No data for IS Peak Name*

**Data File**	2024 12 06 STD AA.wiff	**Result Table**	RW2024112111_AA_ZDD_2024 12 12.rdb
**Acquisition Date**	12/6/2024 7:47:21 PM	**Algorithm Used**	MQL
**Acquisition Method**	Amino acid_Test_YJX_20240827.dam	**Instrument Name**	AB SCIEX QTRAP 5500
**Project**	Products		

Regression Equation: y = 1.49e+006 x (r = 0.9976)

Expected Concentration	Number of Values	Mean Calculated Concentration	% Accuracy	Std. Deviation	%CV
0.01	1	0.01	114.8	NaN	NaN
0.05	1	0.05	107.1	NaN	NaN
0.1	1	0.10	97.7	NaN	NaN
1	1	0.96	95.6	NaN	NaN
5	1	4.78	95.6	NaN	NaN
10	1	10.18	101.8	NaN	NaN
20	1	22.41	112.0	NaN	NaN
50	1	47.67	95.3	NaN	NaN

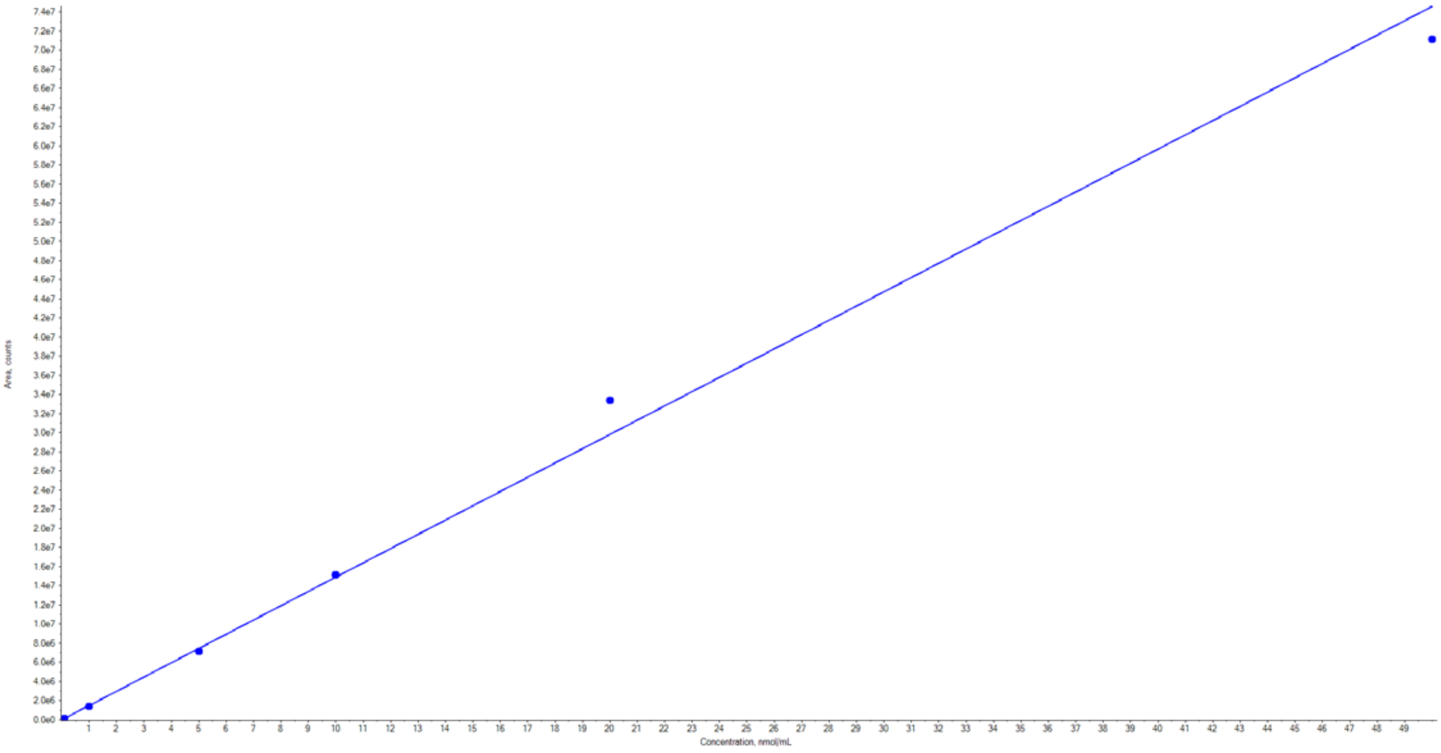

Analyte Name: His_1
Internal Standard: *No data for IS Peak Name*

**Data File**	2024 12 06 STD AA.wiff	**Result Table**	RW2024112111_AA_ZDD_2024 12 12.rdb
**Acquisition Date**	12/6/2024 7:47:21 PM	**Algorithm Used**	MQL
**Acquisition Method**	Amino acid_Test_YJX_20240827.dam	**Instrument Name**	AB SCIEX QTRAP 5500
**Project**	Products		

Regression Equation: y = 5.23e+006 x (r = 0.9995)

Expected Concentration	Number of Values	Mean Calculated Concentration	% Accuracy	Std. Deviation	%CV
0.01	1	0.01	91.6	NaN	NaN
0.05	1	0.05	107.3	NaN	NaN
0.1	1	0.10	98.4	NaN	NaN
1	1	0.96	96.0	NaN	NaN
5	1	4.85	97.0	NaN	NaN
10	1	9.63	96.3	NaN	NaN
20	1	20.56	102.8	NaN	NaN

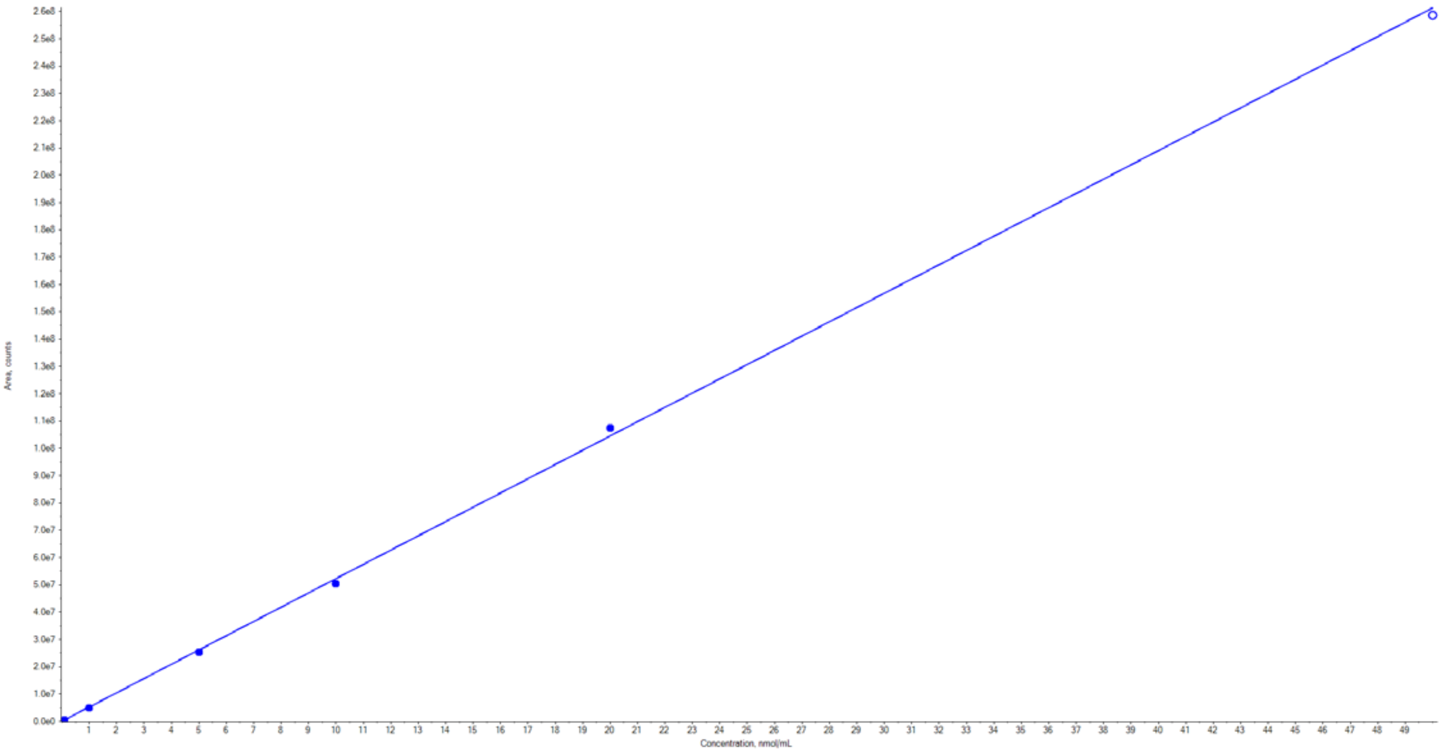

Analyte Name: Pro_2
Internal Standard: *No data for IS Peak Name*

**Data File**	2024 12 06 STD AA.wiff	**Result Table**	RW2024112111_AA_ZDD_2024 12 12.rdb
**Acquisition Date**	12/6/2024 7:47:21 PM	**Algorithm Used**	MQL
**Acquisition Method**	Amino acid_Test_YJX_20240827.dam	**Instrument Name**	AB SCIEX QTRAP 5500
**Project**	Products		

Regression Equation: y = 1.64e+005 x (r = 0.9972)

Expected Concentration	Number of Values	Mean Calculated Concentration	% Accuracy	Std. Deviation	%CV
0.01	1	0.01	105.4	NaN	NaN
0.05	1	0.05	109.7	NaN	NaN
0.1	1	0.13	130.1	NaN	NaN
1	1	1.06	105.7	NaN	NaN
5	1	5.65	113.0	NaN	NaN
10	1	11.03	110.3	NaN	NaN
20	1	21.33	106.6	NaN	NaN
50	1	46.90	93.8	NaN	NaN

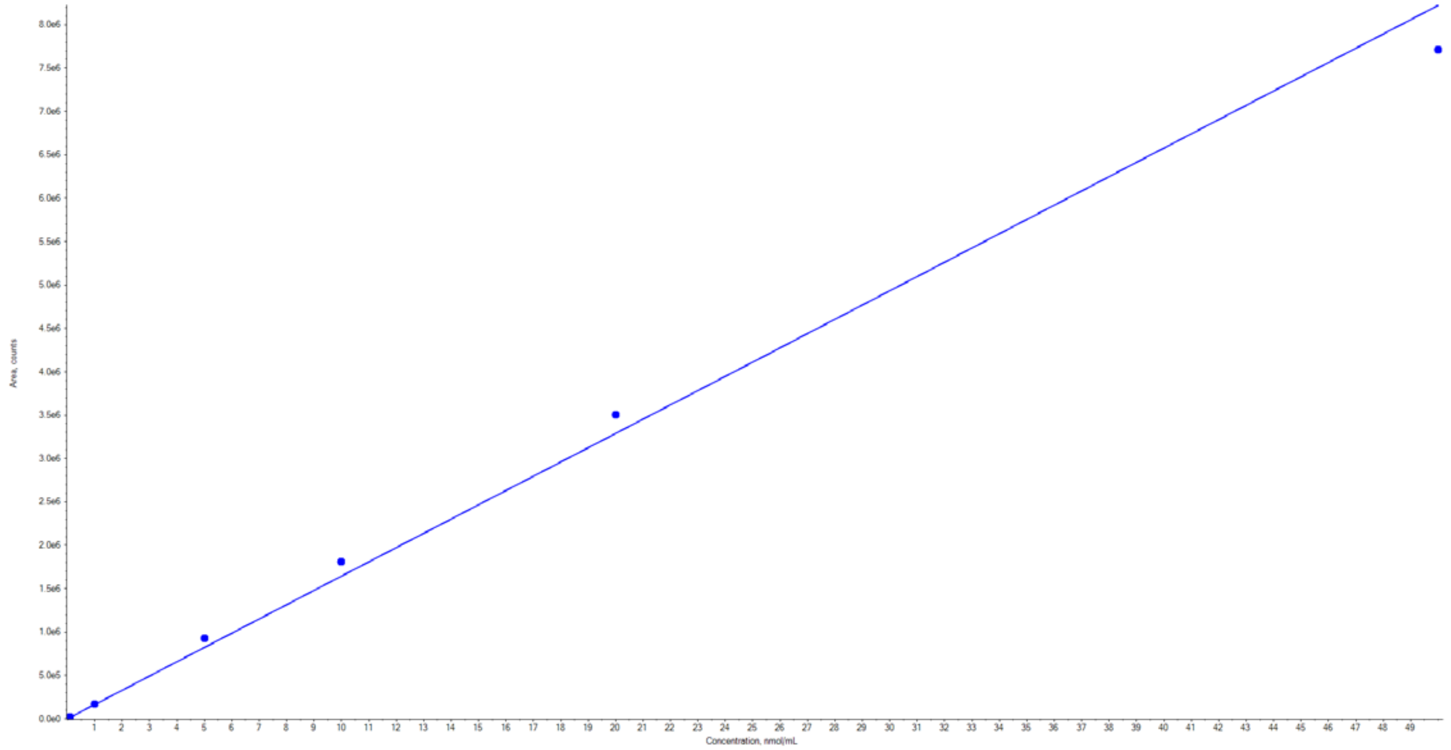

Analyte Name: Met_1
Internal Standard: *No data for IS Peak Name*

**Data File**	2024 12 06 STD AA.wiff	**Result Table**	RW2024112111_AA_ZDD_2024 12 12.rdb
**Acquisition Date**	12/6/2024 7:47:21 PM	**Algorithm Used**	MQL
**Acquisition Method**	Amino acid_Test_YJX_20240827.dam	**Instrument Name**	AB SCIEX QTRAP 5500
**Project**	Products		

Regression Equation: y = 2.37e+006 x (r = 0.9999)

Expected Concentration	Number of Values	Mean Calculated Concentration	% Accuracy	Std. Deviation	%CV
0.01	1	0.01	93.1	NaN	NaN
0.05	1	0.04	87.5	NaN	NaN
0.1	1	0.09	86.9	NaN	NaN
1	1	0.97	96.5	NaN	NaN
5	1	5.02	100.4	NaN	NaN
10	1	10.21	102.1	NaN	NaN
20	1	19.82	99.1	NaN	NaN

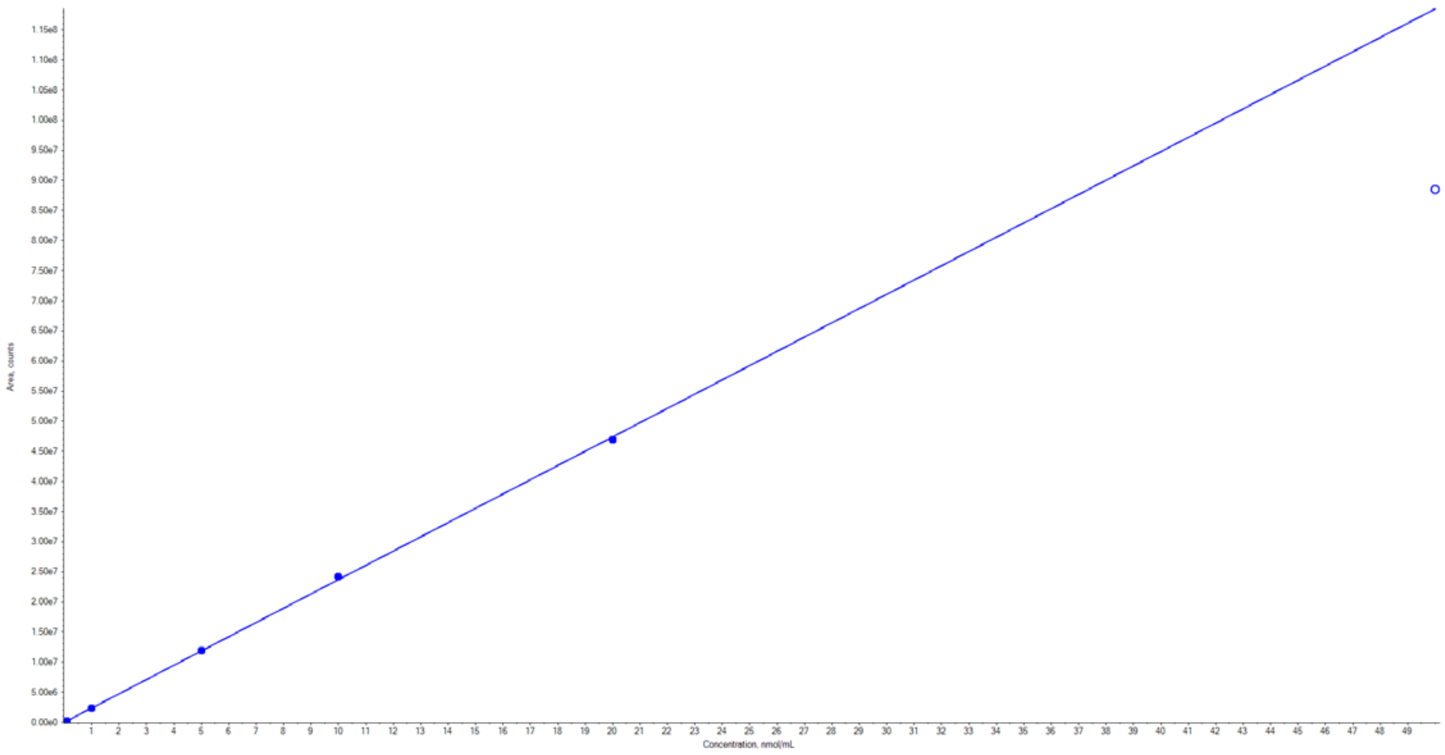

Supplement: Supplementary file 1 [file jof-11-00365-s001.zip › Figure S1.pdf]
