# Supplementary figures and images for: Integrated Amino Acid Profiling and 4D-DIA Proteomics Reveal Protein Quality Divergence and Metabolic Adaptation in Cordyceps Species
Source: J Fungi (Basel). 2025 May 8;11(5):365. doi: 10.3390/jof11050365 (PMC12113033; doi:10.3390/jof11050365)

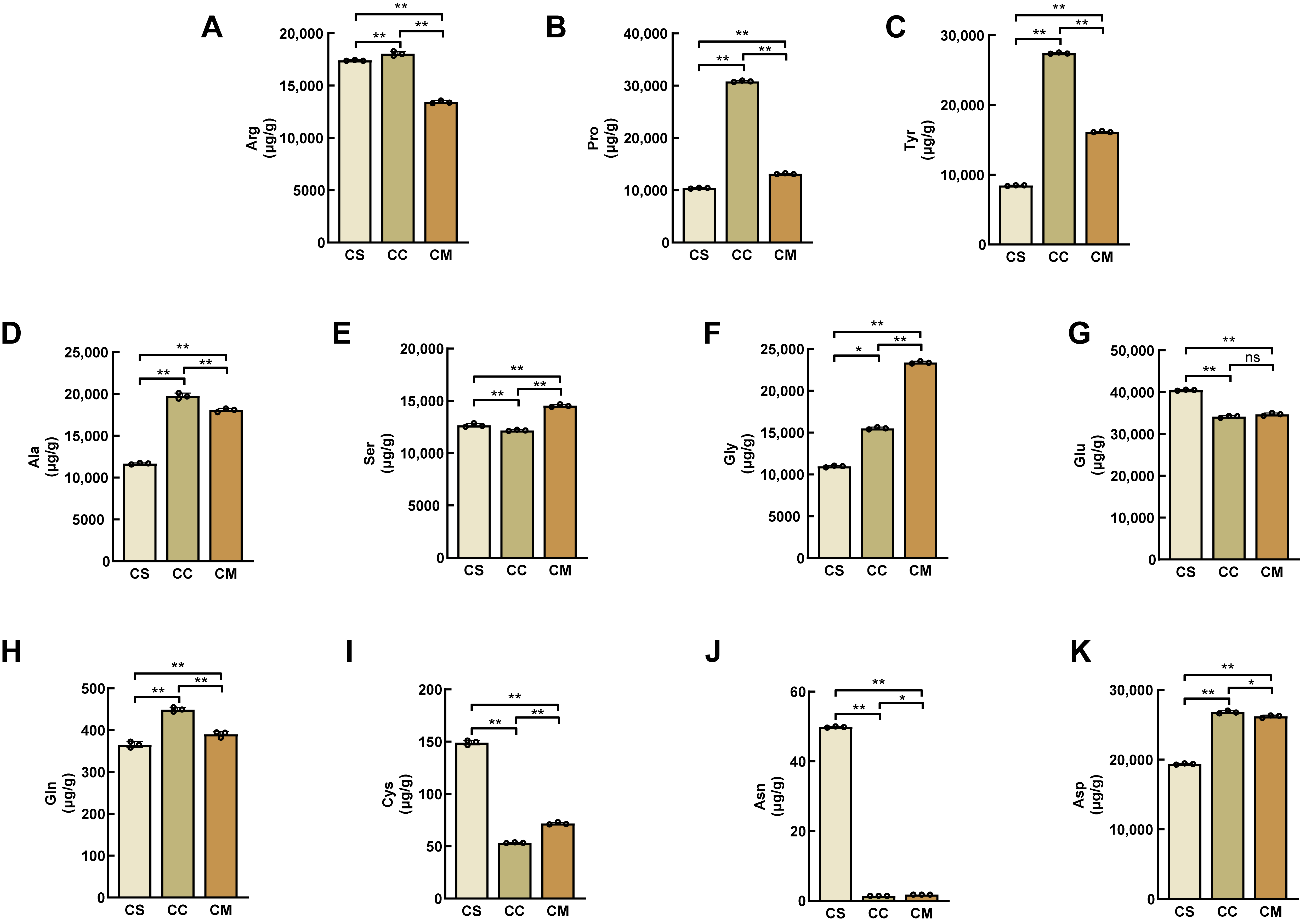

Supplement: Supplementary file 1 [file jof-11-00365-s001.zip › Figure S2.tif]
